# Optimizing selectivity of the Cerebellar Cognitive Affective Syndrome Scale by use of correction formulas, and validation of its German version

**DOI:** 10.1007/s00415-025-13083-3

**Published:** 2025-04-17

**Authors:** Andreas Thieme, Kerstin Rubarth, Raquel van der Veen, Johanna Müller, Jennifer Faber, Miriam Barkhoff, Martina Minnerop, Saskia Elben, Dana Huvermann, Friedrich Erdlenbruch, Adam M. Berlijn, Patricia Sulzer, Kathrin Reetz, Imis Dogan, Heike Jacobi, Julia-Elisabeth Aktories, Giorgi Batsikadze, Qi Liu, Benedikt Frank, Martin Köhrmann, Elke Wondzinski, Mario Siebler, Jürgen Konczak, Matthis Synofzik, Thomas Klockgether, Frank Konietschke, Sandra Röske, Dagmar Timmann

**Affiliations:** 1 Department of Neurology and Center for Translational Neuro- and Behavioral Sciences (C-TNBS), Essen University Hospital, University of Duisburg-Essen, Hufelandstraße 55, 45147 Essen, Germany; 2 Institute of Biometry and Clinical Epidemiology, Charité University Medicine Berlin, Corporate Member of Freie University Berlin, Berlin, Germany; 3 German Center for Neurodegenerative Diseases (DZNE), Bonn, Germany; 4 Department of Neurology, Bonn University Hospital, Rheinische Friedrich-Wilhelms University Bonn, Bonn, Germany; 5 Department of Neurology, Center for Movement Disorders and Neuromodulation, Medical Faculty, Heinrich-Heine University Düsseldorf, Düsseldorf, Germany; 6 Institute of Clinical Neuroscience and Medical Psychology, Medical Faculty, Heinrich-Heine University Düsseldorf, Düsseldorf, Germany; 7 Institute of Neuroscience and Medicine (INM- 1), Research Center Jülich, Jülich, Germany; 8 Faculty of Mathematics and Natural Sciences, Heinrich-Heine University Düsseldorf, Düsseldorf, Germany; 9 Department of Neurodegenerative Diseases, Hertie-Institute for Clinical Brain Research and Center of Neurology, Eberhard-Karls University Tübingen, Tübingen, Germany; 10 German Center for Neurodegenerative Diseases (DZNE) Tübingen, Helmholtz Association, Tübingen, Germany; 11 JARA-BRAIN Institute, Molecular Neuroscience and Neuroimaging, Research Center Jülich, Jülich, Germany; 12 Department of Neurology, Aachen University Hospital, Rheinisch-Westfälische Technische Hochschule (RWTH) Aachen, Aachen, Germany; 13 Department of Neurology, Heidelberg University Hospital, Ruprecht-Karls University Heidelberg, Heidelberg, Germany; 14 Department of Neurology and Neurorehabilitation, MediClin Rhein/Ruhr, Essen, Germany; 15 School of Kinesiology, University of Minnesota, Minneapolis, USA; 16 Berlin Institute of Health (BIH), Berlin, Germany

**Keywords:** Cerebellum, Cognition, Affect, CCAS, CCAS-Scale

## Abstract

**Background:**

Cerebellar disease may result in *Cerebellar Cognitive Affective Syndrome (CCAS)*. The CCAS-Scale, designed to screen for CCAS, has been validated in English Hoche (Brain 141:248–270, 2018) and adapted to other languages.

**Methods:**

Here, the German CCAS-Scale Thieme (Neurol Res Pract 2:39, 2020) was validated in 209 patients with cerebellar disorders and 232 healthy controls. Correction formulas for the outcome parameters [failed test items (range: 1–10) and sum raw score (range: 0–120)] were developed, controlling for age, education, and sex effects. Diagnostic accuracy and reliability were assessed.

**Results:**

Correction formulas improved selectivity in controls, reducing false positives (failed items: 40%; sum score: 13% vs. original method Hoche (Brain 141:248–270, 2018): 67%), while maintaining moderate sensitivity (failed items: 69%; sum score: 48% vs. original method Hoche (Brain 141:248–270, 2018): 87%). Word fluency tests differentiated best between patients and controls, while other items did not. Internal consistency (α = 0.71) was acceptable. Removal of word fluency tests worsened it. Retest and interrater reliability were high [intraclass correlation coefficients (ICC): 0.77–0.95]. However, these ICCs yielded a large minimal detectable change (MDC; 2.2–2.4 failed items, 9.5–11.4 raw score points) in patients, limiting the use of the CCAS-Scale in follow-up examinations.

**Conclusion:**

The correction formulas improved diagnostic accuracy of the CCAS-Scale, particularly for the sum raw score. Therefore, we recommend using the *corrected* sum raw score for evaluation instead of the *uncorrected* number of failed items, proposed originally Hoche (Brain 141:248–270, 2018). Some test items, however, did not differentiate well between patients and controls and MDCs were large, highlighting the need for refined CCAS assessment instruments as progression or treatment outcomes.

**Supplementary Information:**

The online version contains supplementary material available at 10.1007/s00415-025-13083-3.

## Introduction

Cerebellar disease results in cerebellar ataxia [1], often associated with additional cognitive and neuropsychiatric symptoms [2–4]. These non-motor symptoms are referred to as *Cerebellar Cognitive Affective/Schmahmann Syndrome (CCAS)*. Patients with CCAS usually show impairments in the cognitive domains of executive, linguistic, and/or visuospatial functions [5]. The common neuropsychiatric symptoms encompass problems with emotional control and autism- or psychosis-like symptoms as well as difficulties in maintaining adequate social behavior [6].

A standardized test, specifically designed to screen for the cognitive profile present in cerebellar disease (that is: CCAS), was first established in (US-American) English in 2018 by Hoche and colleagues [7]. Their *Cerebellar Cognitive Affective/Schmahmann Syndrome Scale (CCAS-S)* is a brief assessment which can be applied in 10–15 min. The CCAS-S is now in widespread use and makes cognitive assessment of cerebellar patients easier, faster, and more standardized. This enables multicenter studies with larger sample sizes and facilitates comparison of test results between sites and studies. After the initial validation of the original US-American version [7], the CCAS-S has been translated into different languages, including German [8], Spanish [9], Portuguese [10], Hungarian [11], Dutch, and French [12].

Although the CCAS-S has some limiting drawbacks, such as a high rate of false-positive results in healthy controls, partly caused by sex, age, and education effects [13, 14], the CCAS-S has been used in several studies, including patients with autosomal-dominant ataxias [9, 13, 15, 16], autosomal-recessive ataxias [13, 17, 18], and cerebellar stroke [19, 20]. Moreover, it has been recommended for use in clinical trials [21], despite its limitations. The increasing application of the CCAS-S in multinational, multicenter clinical trials, makes validation of all translated versions of the CCAS-S necessary. Here, we present data of the validation of the German version. In addition, we introduce correction formulas which account for the known sex, age, and education effects [14] on CCAS-S performance.

## Methods

### Participants

Participants were recruited at the University Hospitals of Aachen, Bonn, Düsseldorf, Essen, Heidelberg, and Tübingen in Germany. In total, 245 patients (205 with degenerative cerebellar disease and 40 with cerebellar stroke) and 265 controls participated after giving their written informed consent. Thirty-six patients and 33 controls were excluded, because they fulfilled exclusion criteria (see Table S1, supplementary material).

The final data set consisted of 209 patients (188 with degenerative disorder and 21 isolated cerebellar stroke) and 232 healthy controls. Some participants from the current study sample were included in preliminary analyses conducted by our research group (107 patients and 97 controls in [14], and 64 patients and 64 controls in [13]).

Based on medical history and neurological examination, none of the participants in the final data set suffered from psychiatric or neurological disorders other than cerebellar degeneration or isolated cerebellar stroke. Patients’ characteristics are shown in Table S2, supplementary material. All participants were native German speakers.

The study was approved by the local ethics committees and conforms to the Declaration of Helsinki. The study has been prospectively registered at the German Clinical Study Register (https://www.drks.de; DRKS-ID: DRKS00016854).

### Cerebellar Cognitive Affective Syndrome Scale

The CCAS-S consists of 12 test items. Performance in ten items is scored: *semantic* and *phonematic word fluency*, *category switching*, *digit span forward* and *backward*, *cube drawing*, *delayed verbal recall*, *similarities*, *go/no-go*, and *affect* (for details, see supplementary materials, Table S3 as well as [7] and [8]). Each test item has a raw score. Based on item-specific thresholds introduced in the US-American validation study [7] an item is either passed or failed. According to Hoche et al. [7], the presence of CCAS is considered *possible* if one item is failed, *probable* if two items are failed, and *definite* if three or more items are failed. Additionally, a total sum raw score can be calculated (range: 0–120) by summation of the single items’ raw scores. Hoche and colleagues recommended to use the total sum raw score only for intra-individual comparison in follow-examinations, but not to determine whether an individual is diagnosed with CCAS or not.

### Assessment of ataxia, non-ataxia signs, and demographic and clinical variables

For the purpose of correlation analysis of cognitive performance with other symptoms, different clinical rating scales were applied. The Scale for the Assessment and Rating of Ataxia (SARA) was used to rate the severity of ataxia in all patients [22]. In a subgroup, the International Cooperative Spinocerebellar Ataxia Rating Scale (ICARS) [23] was acquired as an additional measure of ataxia severity. The Inventory of Non-Ataxia Signs (INAS) [24] count served as a semiquantitative measure of non-ataxia signs allowing to estimate the degree of extracerebellar involvement.

Moreover, age (in years, yrs), educational level (i.e., yrs of primary + secondary + tertiary education), and sex category were assessed in all participants. Additionally, disease duration (in yrs; i.e., time interval between onset of ataxia and study inclusion) and (molecular) diagnosis were registered for further correlation analyses in patients.

The methodology and the results of the correlation analyses are found in the supplementary materials, M1.

### Validation of the German Cerebellar Cognitive Affective Syndrome Scale

Validation followed guidelines for cross-cultural adaptation and validation of scales in healthcare research [25, 26]. In brief, the process involved several steps of translations and backtranslations of the four original US-American CCAS-S versions A-D involving German neuroscientists and ataxia experts (M. Synofzik, P. Sulzer, J. Faber, S. Röske, J. Konczak, D. Timmann, and A. Thieme), being fluent in English (J. Konczak was bilingual), as well as the senior author of the US-American CCAS-S (J.D. Schmahmann). A preliminary consensus version of the German CCAS-Scale (G-CCAS-S) was then pretested in a small group of German-speaking patients, healthy controls, and healthcare providers to ensure that the G-CCAS-S used clear and unequivocal test instructions. Where necessary, minor adaptations were made prior to the actual validation [8].

In the current validation study, prior to any assessments, all investigators were requested to study the detailed test manual published in the supplementary material of Thieme et al. [8]. Version A of the G-CCAS-S was then administered to each participant at least once. A subset of patients and healthy controls received a follow-up examination with the same version of the scale to determine test–retest (i.e., intrarater; 21 patients, 22 controls) and interrater (19 patients, 23 controls) reliability. Follow-up examinations took place within an interval of 7–49 days. A time interval was favored instead of a fixed time span (e.g., exactly 14 days) between baseline (T1) and retest (T2) to control for possible learning/training effects. For intrarater reliability, a participant was examined in both test sessions (T1 and T2) by the same rater, and for interrater reliability by different raters.

Moreover, as in the study by Hoche et al. (2018), the Montreal Cognitive Assessment (MoCA) was assessed in a subset of 26 patients and 24 controls to investigate if the G-CCAS-S is more sensitive and selective than the German MoCA to detect cognitive deficits in cerebellar patients. Hoche et al. have shown for the US-American CCAS-S that patients’ total MoCA scores were within in the same range as those of healthy controls, while the CCAS-S scores were not. These findings were explained by the different designs of both assessments. While the MoCA focusses on memory deficits and is optimized to screen for dementia, the CCAS-S focusses on executive dysfunction and is optimized to screen for CCAS [7].

### Development of correction formulas

Previous studies of our [13, 14] and other groups [9, 16] have shown that cognitive performance measured by the CCAS-S depends on age and educational level, and to a lesser degree on sex. This contributed to a high rate of false-positive results in healthy controls. Neglecting these effects, the CCAS-S might overestimate the presence of a CCAS also in patients. This study confirmed the presence of age, education, and sex effects. To encounter these effects, we developed correction formulas for the number of failed test items and the total sum raw score.

First, control participants who failed the *verbal recall* (*n* = 29) and/or the *affect* (*n* = 3, whereof 2 also failed *verbal recall*) test item were excluded from regression analysis for developing correction formulas. This step was conducted to ensure a baseline of optimal cognitive performance in healthy controls. Failure of the *verbal recall* item might indicate mild cognitive impairment and failure on the item *affect* mild emotional dysfunction.

For model training, 80% of the remaining controls were randomly selected, and the models were fitted using this subset. Subsequent evaluation, involving sensitivity and selectivity calculations, was performed in the remaining 20% of the controls and all patients with cerebellar degeneration. This partitioning facilitated an independent assessment of the models, using separate training and evaluation datasets.

Following this, two regression models were fitted: a Poisson regression model for the number of failed test items and a multiple linear regression model for the total sum raw score. Overdispersion was checked and found to be absent for the number of failed test items. These models were adjusted for sex, age, and educational years in healthy controls.

Regarding failed test items, the predicted number of failed test items was calculated for each patient in the evaluation dataset and compared to the actual number of failed items. If a patient’s actual exceeded the predicted number of failed test items, the patient was labeled as *abnormal*; if the actual number was equal to or smaller than the predicted number, the patient was labeled as *normal*. Sensitivity and selectivity were calculated. To assess sensitivity, the percentage of true positives was calculated, that is the percentage of patients who were correctly identified as patients (number of patients identified as patients/true number of patients in the sample* 100). To assess selectivity, the percentage of true negatives was calculated, which is the percentage of controls that have been correctly identified as controls (number of controls identified as controls/true number of controls in the sample* 100). Moreover, the rate of false positives was calculated in controls (100%—selectivity) [14].

Because the range of possible values was wider for the total sum raw score (range: 0–120) compared to the number of failed test items (range: 0–10), 1-α prediction intervals were calculated for the total sum raw score for each patient in the evaluation dataset. Patients were labeled as *normal* if their observed total sum raw score exceeded the lower bound of the prediction interval, and as *abnormal* if it was lower than this threshold. Sensitivity, selectivity, and false-positive rate were then calculated for various α-levels and compared.

### Application of correction formulas

Next, the correction formulas were applied to the whole sample of patients and controls and to subgroups of patients with a specific diagnosis. Sensitivity and selectivity were assessed as described above and compared to sensitivity and selectivity computed using the method of Hoche et al. [7]. The percentage of patients and controls being categorized as CCAS *absent* (0 items failed), *possible* (1 item failed), *probable* (2 items failed), and *definite* (≥ 3 items failed) was determined. For each classification category (that is: *CCAS possible/probable/definite*), sensitivity and selectivity as well as the false-positive rate were determined.

### Group comparisons with uncorrected scores

Next, the *uncorrected* number of failed test items, the *uncorrected* total sum raw score, the percentage of failures on single test items, and the single test items’ raw scores were compared between patients and controls. Because age, education, and sex category play a role, and the correction formulas only apply for the overall performance (i.e., total number of failed test items and total sum raw score), a matched-pairs design was used: For each patient, a control was matched for age (± 5 yrs), educational level (± 3 yrs of education), and, if possible, for sex. For 12 of the patients with cerebellar degeneration, no match was found. Therefore, they were excluded from group comparisons. Final matched group characteristics are shown in Table S4, supplementary material.

Results from a Shapiro–Wilk test and histograms showed that the data were not normally distributed (all p values ≤ 0.028). The *uncorrected* number of failed test items, the *uncorrected* total sum raw score and raw scores of single test items were compared using estimation statistics focusing on effect size (in this study the mean difference was used as effect size) rather than solely on significance testing (https://www.estimationstats.com). Permutation t tests were calculated. For each permutation p value, 5000 reshuffles were performed. The permutation t test is robust to non-normal distributions [27]. The percentage of failures on single test items and the total number of failed test items in patients and controls was compared using Fisher’s exact test.

### Receiver-operating curve analyses

To further explore the diagnostic accuracy of the G-CCAS-S, a receiver-operating curve (ROC) analysis was performed in the matched groups graphing the true-positive (sensitivity) versus the false-positive rate (100%—selectivity) considering the *uncorrected* number of failed test items, the *uncorrected* total sum raw score, and single test items’ raw scores. The methodology and the results of ROC analyses are found in the supplementary materials, M2.

### Reliability of the G-CCAS-S

To determine test–retest (intrarater) and interrater reliability, the intraclass correlation coefficient [ICC (absolute agreement), two-way random effects model for both measures, average measures], the standard error of measurement (SEM), and the minimal detectable change at the 80% (MDC80%) and the 95% (MDC95%) confidence interval (CI) were calculated. The SEM quantifies the amount of the error inherent in any measurement due to the limitations of the measurement instrument or the variability in the measurement process. The MDC reflects the smallest real amount of change in a score that exceeds the SEM [28, 29]. The formulas used to calculate SEM, MDC80% and MDC95% are given in the supplementary materials, M3.

Moreover, to check for possible learning effects, the improvement/worsening (Δ) of the overall test measures was calculated as follows:

Δ *uncorrected* failed test items = *uncorrected* failed test items at T2 – *uncorrected* failed test items at T1 and

Δ *uncorrected* total sum raw score = *uncorrected* total sum raw score at T2 – *uncorrected* total sum raw score at T1.

These measures were then correlated with the time interval between the baseline (T1) and retest (T2) using Spearman’s rank correlation coefficient [30]. Our hypothesis was that if learning effects existed, these would be expected to decline with an increasing time interval between T1 and T2.

Next, in accordance with the study by Hoche et al. [7], internal consistency was examined using Cronbach’s alpha. Internal consistency is a measure that expresses the interrelatedness of the single test items. This method enables evaluating if all test items of the G-CCAS-S measure the same concept—in this case: CCAS. Internal consistency is considered poor for a Cronbach’s alpha of < 0.7, acceptable for ≥ 0.7, good for ≥ 0.8, and excellent for ≥ 0.9 [7, 31].

### Comparison of Cerebellar Cognitive Affective Syndrome Scale and Montreal Cognitive Assessment

Next, performance on the G-CCAS-S (version A) and the German MoCA (version 1) were compared in a subgroup of 26 patients and 24 healthy controls. The methodology and the result of this analysis are found in the supplementary materials, M4.

### Statistics

All statistical analyses were performed by use of the programs IBM SPSS Statistics (version 29.0.2.0; https://www.ibm.com/products/spss-statistics), and GraphPad Prism (version 10.4.1; https://www.graphpad.com/scientific-software/prism) as well as the website “estimationstats” (https://www.estimationstast.com). Null hypotheses were rejected for a p value < 0.05. Due to the exploratory nature of the study, no adjustment for multiplicity was conducted, and hence, all analyses were interpreted in a hypothesis-generating manner. All figures were generated using GraphPad Prism (version 10.4.1) and Corel Draw Graphics Suite 2021 (version 23.1.0.389; https://www.coreldraw.com/de/product/coreldraw/).

## Results

### Development of correction formulas

Because of the known age, education, and (to a lesser degree) sex effects [9, 14, 16] which have been replicated in this study (Table S5 and S6, supplementary material), correction formulas were developed based on performance in healthy controls (Table 1).

**Table 1:** Poisson regression model for the number of failed test items and linear regression model for the total sum raw score

Failed test items					
Factor	Risk ratio ± SE	*z* value	*p* value	Lower bound of 95% CI	Upper bound of 95% CI
Intercept	2.70 ± 0.43	2.31	0.02	1.16	6.28
Female	0.88 ± 0.13	− 0.98	0.33	0.67	1.14
Age (yrs)	1.01 ± 0.00	3.67	0.00	1.01	1.02
Education (yrs)	0.91 ± 0.02	− 4.25	0.00	0.87	0.95

Total sum score					
Factor	Estimate ± SE	*t* value	*p* value	Lower bound of 97.5% CI	Upper bound of 97.5% CI
Intercept	93.3 ± 3.37	27.70	0.008	6.66	99.94
Female	2.93 ± 1.13	2.60	0.01	0.70	5.15
Age (yrs)	− 0.12 ± 0.03	− 3.85	0.00	− 0.18	− 0.06
Education (yrs)	0.76 ± 0.17	4.44	0.00	0.42	1.10

*SE* standard error, *CI* confidence interval, *yrs* years

The Poisson regression model for the number of failed test items revealed that the risk of failing items increases by ~ 1% per year of life and it decreases by ~ 9% per year of education. Moreover, there was a trend indicating that female sex decreases the risk of failing a test item (risk ratio: 0.88).

The linear regression model for the total sum raw score revealed that females score on average 2.93 points higher than men. The total sum raw score decreases with ~ 0.12 total points per year of life and increases by ~ 0.78 per year of education.

The derived correction formulas to predict the number of failed test items Y (Eq. 1) or the lower bound of the prediction interval for the total sum raw score Z (Eq. 2) are shown below:

Predicted failed test items:

1 $${\text{Y }} = {\text{ e}}^{{(0.{99 } - \, 0.{13 }*{\text{ sex }} + \, 0.0{1 }*{\text{ age }} - \, 0.{1 }*{\text{ edu}})}} .$$

Predicted lower bound of total sum raw score:

2 $$Z = 93.3 + 2.92 \times {\text{sex}} {-} 0.12 \times {\text{age}} + 0.76 \times {\text{edu}} {-} z1 - \alpha /2 \times 7.93 \times \sqrt {1.18 - 0.02 \times {\text{edu}},}$$

where *sex* is 0 if participant is a male, and *sex* is 1 if participant is a female; *age* is the age of the participant in years; *edu* is the educational years of the participant.

If a participant reaches a number of failed test items that is greater than the predicted number of failed test items Y (Eq. 1) or a total sum raw score that is below the lower bound Z of the prediction interval of the total sum raw score, the test result is considered *abnormal* (i.e., pathological).

Validation of our correction formulas was performed by application to the evaluation data set (20% of the controls and all patients with cerebellar degeneration). Results for sensitivity and selectivity calculated for various delta, respectively, 1-α levels are shown in Table 2.

**Table 2:** Sensitivity and selectivity for number of failed test items at various delta levels and for total sum raw score at various α levels

Failed test items		
Difference [Δ = predicted failed test items—actual failed test items]	Sensitivity [%]	Selectivity [%]
0	59	68
1	40	88
2	26	96

Total sum score		
1-α level	Sensitivity [%]	Selectivity [%]
0.60	61	77
0.70	56	81
0.80	49	86
0.90	44	92
0.95	40	95

### Application of correction formulas

Next, we applied the correction formulas to our whole sample of patients and controls, as well as subgroups of patients. The classification as *normal/abnormal* test result based on the correction formulas was then compared to the categorization according to Hoche et al. [7] in terms of sensitivity, selectivity, and false positives.

#### All patients and controls

Considering the number of failed test items, 69% of all patients were considered to have an *abnormal* test when using the correction formula. In contrast, according to the *uncorrected* method of Hoche et al. [7], 87% of the patients were considered to have an *abnormal* test to some extent (CCAS *possible/probable/definite*: 21/20/46%).

**Fig. 1:**
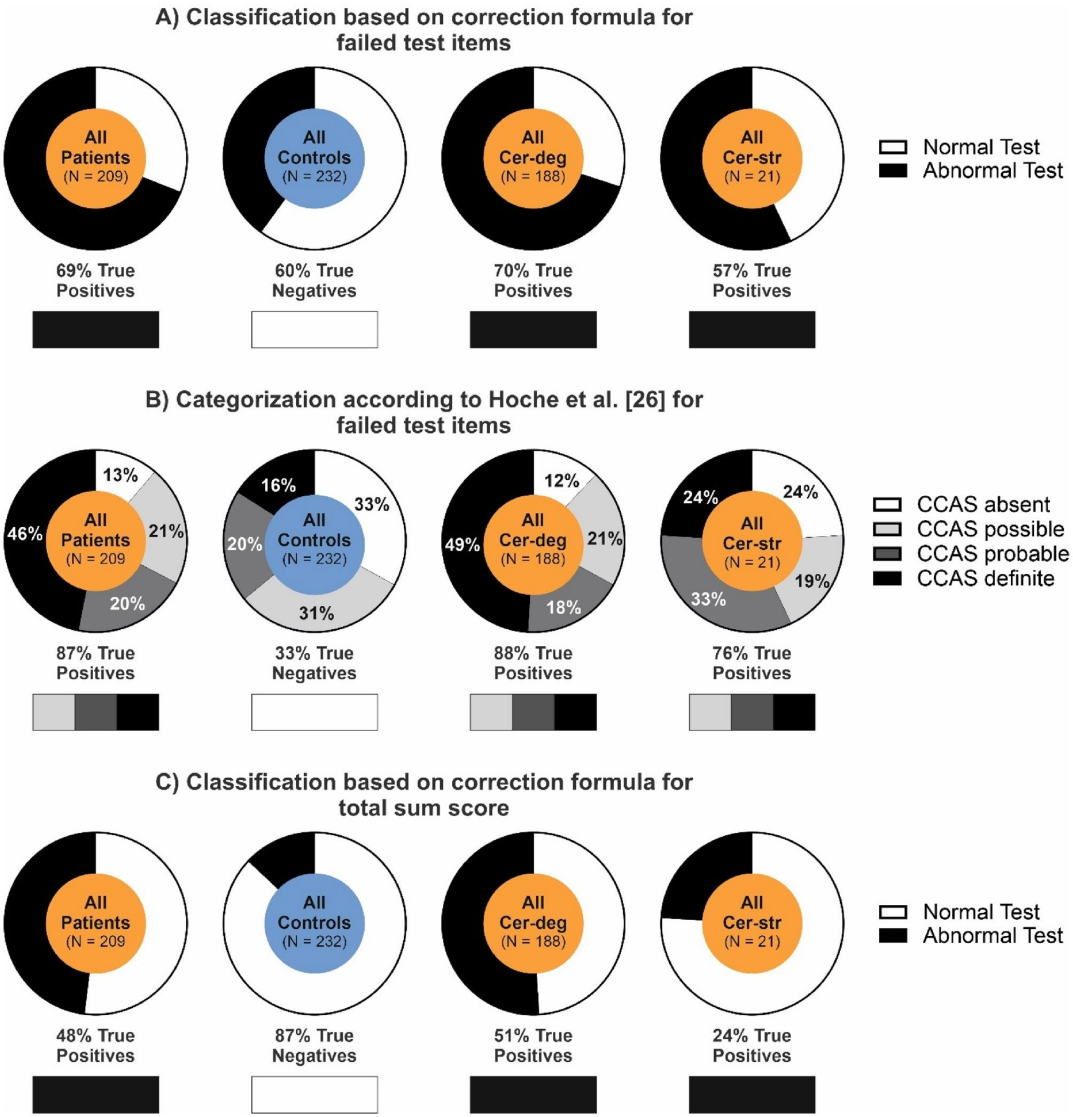
Classification as *normal/abnormal* test result based on newly developed correction formulas in comparison to the original categorization as *CCAS possible/probable/definite* according to Hoche et al. [7]. **A** Classification as *normal/abnormal* test result based on the newly developed correction formula for the number of failed test items. **B** Categorization as *CCAS possible/probable/definite* according to Hoche et al. [7]. **C** Classification as *normal/abnormal* test result based on the newly developed correction formula for the total sum raw score. **A**–**C** Bars below pie charts indicate the percentage of true-positive test results in patients (i.e., the portion of patients that has been correctly identified as patients) and true-negative test results in controls (i.e., the portion of controls that has been correctly identified as controls) based on the different methods for test interpretation

Considering the subgroups of patients, a *(corrected) abnormal* test was present more often in patients with cerebellar degeneration (70%, 132/188) compared to patients with cerebellar stroke (57%, 12/21). Using the *uncorrected* evaluation criteria of Hoche et al. yielded *abnormal* test results in 88% (CCAS *possible/probable/definite*: 21/18/49%) of the patients with cerebellar degeneration and 76% (19/33/24%) with cerebellar stroke (Fig. 1A and B).

Sensitivity (i.e., true positives) was 69% using the correction formula (70% cerebellar degeneration vs. 57% cerebellar stroke). Using the *uncorrected* evaluation criteria by Hoche et al. yielded a sensitivity for *possible/probable/definite* CCAS of 87/67/47% in all patients (cerebellar degeneration: 88/68/50%, cerebellar stroke: 76/57/24%). The classification as a *normal/abnormal* test result based on the correction formula for the total sum raw score is shown in Fig. 1C.

Selectivity (true negatives) was 60% using the correction formula and 33/64/84% for the *uncorrected* categorization CCAS *possible/probable/definite* according to Hoche and colleagues [7]. Hence, using the correction formula, false-positive results in controls were reduced to 40% compared to 67% using the *uncorrected* method (31% of all controls failed ≥ 1 item, 20% failed ≥ 2 items, and 16% failed ≥ 3 items).

Using the correction formula for the total sum raw score at the 95% CI level yielded less sensitive, but more selective test results (Fig. 1C).

#### Subgroups of patients with specific diagnoses

Subgroups of (at least ten) patients with a specific diagnosis were analyzed separately to gain insight if the CCAS-S has differential diagnostic abilities in different forms of ataxia. According to that, data were available for Friedreich’s ataxia (FRDA, *n* = 22), spinocerebellar ataxias type 3 (SCA3, *n* = 45), type 6 (SCA6, *n* = 18), type 14 (SCA14, *n* = 12), and strokes within the territory of the posterior inferior cerebellar artery (PICA stroke, *n* = 17, whereof: 9 left-, 7 right-, and 1 both-sided).

Patients with FRDA (1.7 ± 1.5) failed on average the least test items (*uncorrected*) and had the highest *uncorrected* total sum raw score (95.1 ± 10.9). Patients with SCA3 (2.2 ± 1.7), with SCA14 (2.0 ± 1.7) and with PICA stroke (2.2 ± 2.1) failed more test items (*uncorrected*) and had lower *uncorrected* total sum raw scores (SCA3: 90.6 ± 12.1; SCA14: 92.5 ± 13.1; PICA stroke: 92.2 ± 12.0) than patients with FRDA. Patients with SCA6 failed most test items (2.7 ± 2.3; *uncorrected*), whereas the *uncorrected* total sum raw score (90.6 ± 14.1) was within the same range as in SCA3, SCA14, and PICA stroke patients.

**Fig. 2:**
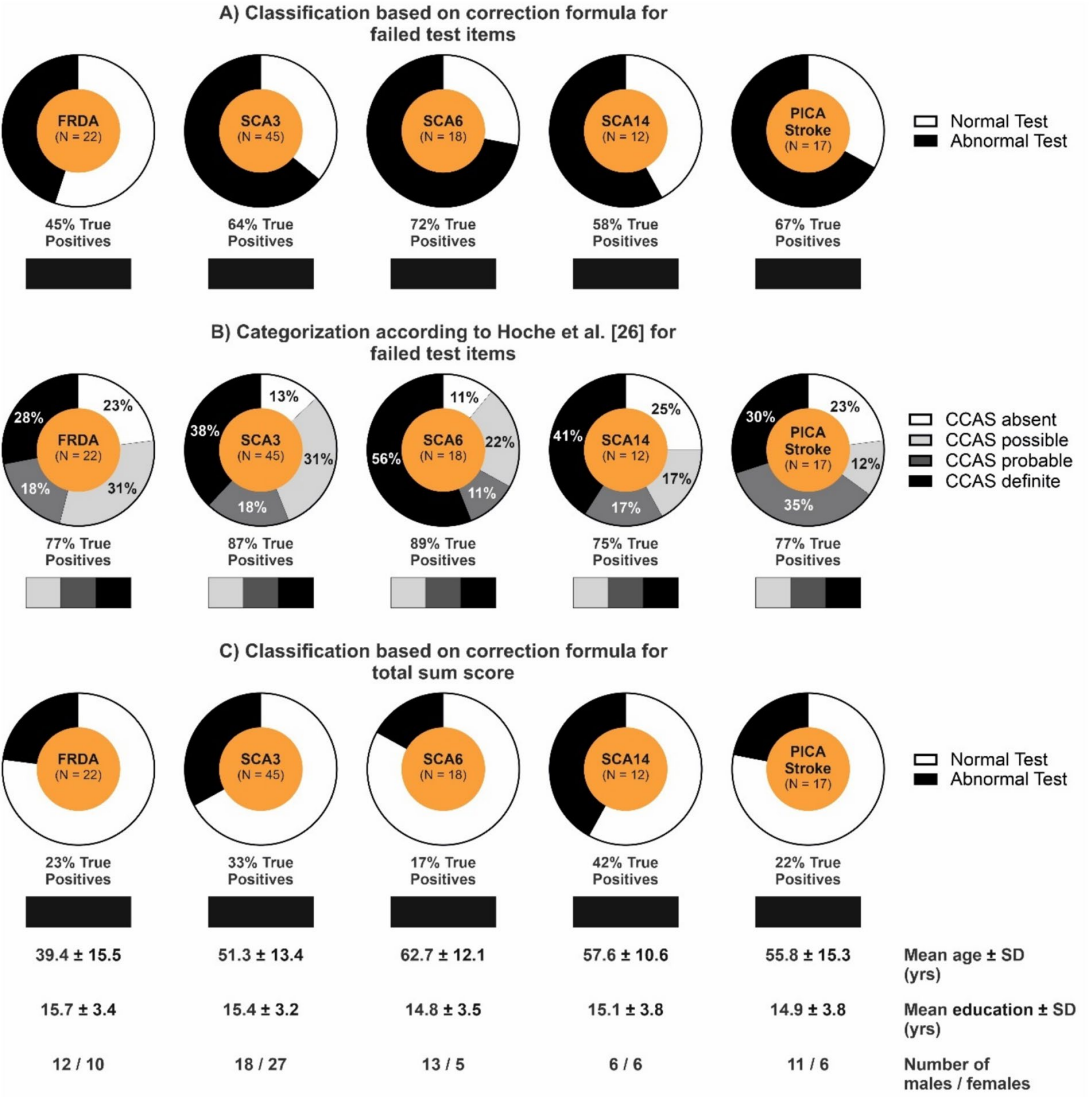
Application of the newly developed correction formulas to subgroups of patients and comparison to the categorization as *CCAS possible/probable/definite* according to Hoche et al. [7]. **A** Classification as *normal/abnormal* test result based on the newly developed correction formula for the number of failed test items. **B** Categorization as *CCAS possible/probable/definite* according to Hoche et al. [7]. **C** Classification as *normal/abnormal* test result based on the correction formula for the total sum raw score. **A**–**C** Bars below pie charts indicate the percentage of true-positive test results in patients (i.e., the portion of patients that has been correctly identified as patients) based on the different methods for test interpretation

Using the correction formula for failed test items, controlling for the different demographics in the subgroups of patients yielded the following percentages of *abnormal* test results: FRDA (45%), SCA3 (64%), SCA6 (72%), SCA14 (58%), and PICA stroke (67%) (Fig. 2A).

Using the correction formula for the total sum raw score, 23% FRDA, 33% SCA3, 17% SCA6, 42% SCA14, and 22% PICA stroke patients were classified to have an *abnormal* total sum raw score (Fig. 2B).

### Group comparisons with uncorrected scores

Next, group comparisons were carried out using the *uncorrected* scores for the number of failed test items, the total sum raw score, single test items’ raw scores, and the percentage of failures on single test items within each group. Because of the known sex, age, and education effects, a matched-pairs design was used.

The group of patients with cerebellar degeneration and the group of patients with cerebellar stroke did not differ significantly regarding age and level of education from their respective matched controls (Table S4, supplementary material; *p* values > 0,121; two-sided permutation *t* test). The sex distribution was identical between patients with cerebellar stroke and their controls and not significantly different between patients with cerebellar degeneration and the respective controls (*p* = 0.201; two-sided Fisher’s exact test).

#### Uncorrected total failed test items

**Fig. 3:**
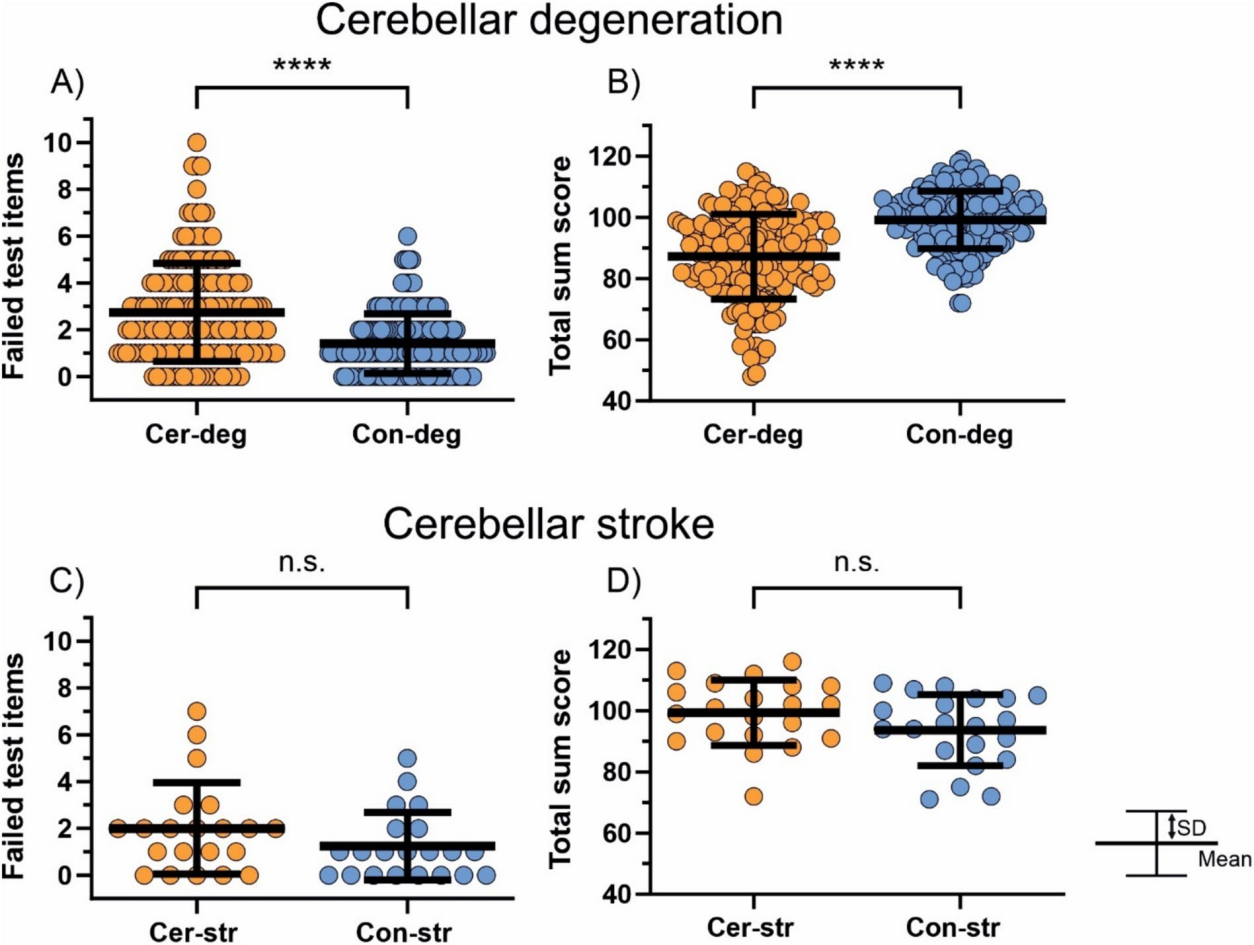
*Uncorrected* total failed test items and total sum raw score. The total number of failed test items and the total sum raw score are shown for patients with cerebellar degeneration (orange; **A**, **B**) and patients with cerebellar stroke (orange; **C**, **D**) and their respective matched controls (blue; **A**–**D**). Each circle represents one participant. Error bars display means and standard deviations. Significant group comparisons are indicated by asterisks. *n.s.* not significant, *SD* standard deviation

Patients with cerebellar degeneration and patients with cerebellar stroke failed on average more test items than their respective controls (Cer-deg: 2.7 ± 2.1 vs. Con-deg: 1.4 ± 1.3, *n* = 176 each; Cer-str: 2.0 ± 1.9 vs. Con-str: 1.2 ± 1.4; *n* = 21 each). The difference between patients with cerebellar degeneration and their controls was significant [unpaired mean difference (MD): 1.3; 95% confidence interval (CI) lower bound, upper bound: 1.0, 1.7; *p* < 0.001, two-sided permutation *t* test; Fig. 3A], while the comparison between patients with cerebellar stroke and their controls did not reach significance [MD: 0.8; CI: − 0.2, 1.8; *p* = 0.137; Fig. 3C].

#### Uncorrected total sum raw score

Patients with cerebellar degeneration and patients with cerebellar stroke had on average a lower total sum raw score than their respective controls (Cer-deg: 87.2 ± 13.9 vs. Con-deg: 99.2 ± 9.4; Cer-str: 93.6 ± 11.7 vs. Con-str: 99.3 ± 10.7). The difference between patients with cerebellar degeneration and their controls was significant [MD: − 12.0; − 14.5, − 9.5; *p* < 0.001, two-sided permutation t test; Fig. 3B], whereas the difference between patients with cerebellar stroke and their controls was not [MD: − 5.7; CI: − 12.4, 0.7; *p* = 0.100; Fig. 3D].

#### Single test items

**Fig. 4:**
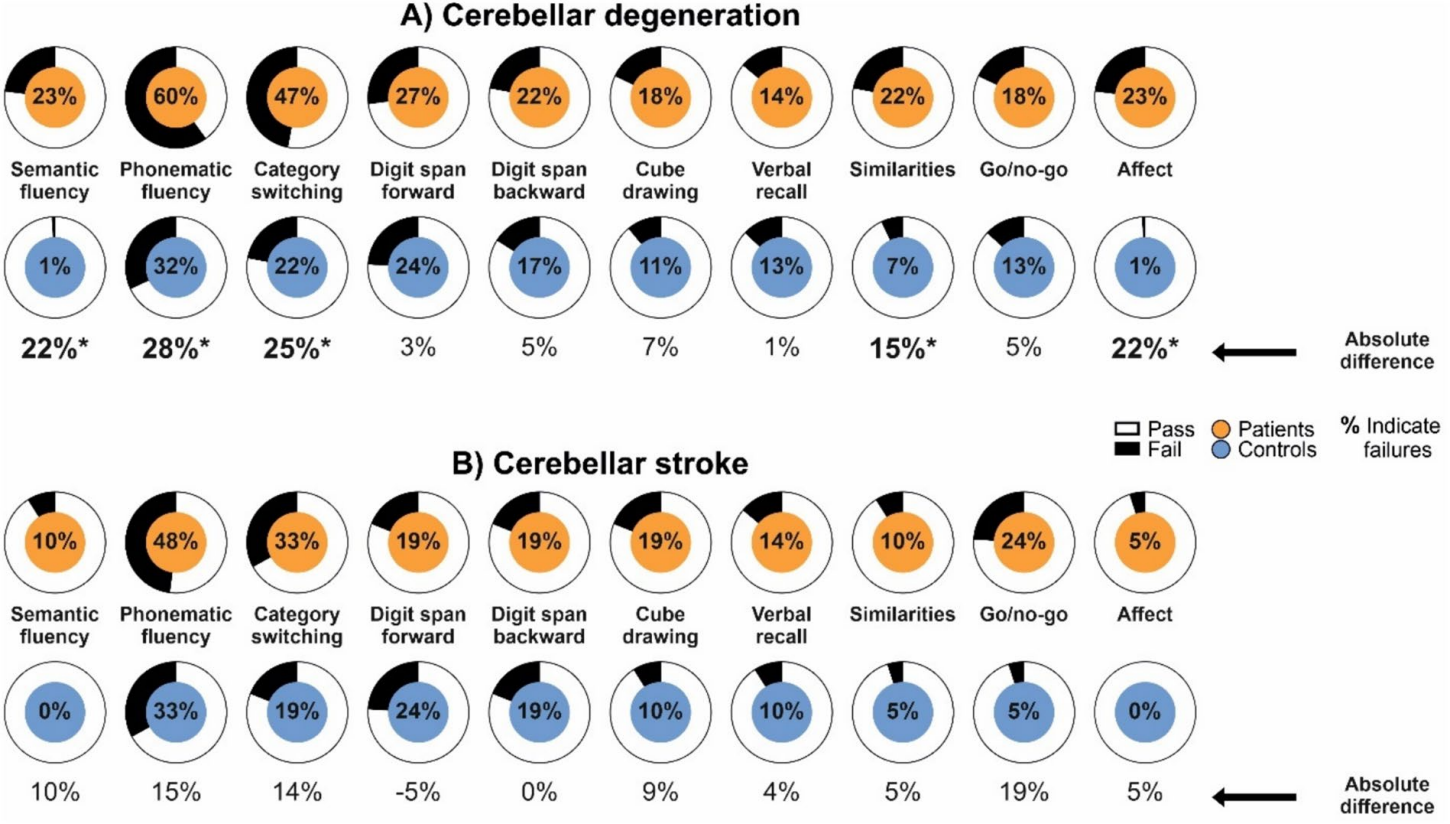
Failures on single test items. Failures on single test items are shown for patients with cerebellar degeneration (orange; **A**) and patients with cerebellar stroke (orange; **B**) and corresponding controls (blue; **A**, **B**). Percentages in the pie charts indicate the percentage of failures within the respective groups. The difference in failure rates (i.e., percentage of failures in patients—percentage of failures in controls) is shown below the pie charts (absolute difference). Based on Fisher’s exact test, failure rates were significantly different between patients with cerebellar degeneration and their corresponding controls on the following test items: *semantic* and *phonematic word fluency*, *category switching*, *similarities*, and *affect*. Significant differences are indicated by an asterisk

Based on pass/fail criteria using the *uncorrected* cut-offs introduced by Hoche et al. [7], the items *semantic fluency* (absolute difference [AD] failures patients – failures controls: 22%), *phonematic fluency* (AD: 28%), *category switching* (AD: 25%), *affect* (AD: 22%), and *similarities* (AD: 15%) showed the largest numerical differences between failures of patients with cerebellar degeneration and matched controls and were statistically significant (*p* values < 0.001; two-sided Fisher’s exact test; Fig. 4A, Table S8, supplementary material). All other items showed numerical differences below 10%.

Between patients with cerebellar stroke and their matched controls, the largest numerical differences were seen on the items *semantic fluency* (AD: 10%), *phonematic fluency* (AD: 15%), *category switching* (AD: 14%), and *go/no-go* (AD: 19%). All other items showed numerical differences below 10%. Statistically significant differences were not present on any test item (p values > 0.184; two-sided Fisher’s exact test; Fig. 4B; Table S8, supplementary material).

Based on *uncorrected* raw scores of single test items, the items *semantic fluency* [mean difference (MD); 95% CI lower bound, upper bound: − 3.7; − 4.6, − 2.89], *phonematic fluency* (MD: − 3.0; − 3.9, − 2.2), and *category switching* (MD: − 2.5; − 3.2, − 1.7) showed the largest numerical differences between patients with cerebellar degeneration and matched controls. Despite statistically significant group differences were present between patients with cerebellar degeneration and their matched controls for all test items except *verbal recall* (*p* values < 0.045, two-sided permutation *t* test; Table S8, supplementary material), these differences were too small to be clinically relevant, except for the above-mentioned word fluency tests. For example, patients with cerebellar degeneration were able to repeat on average 6.1 ± 1.1 digits on the *digit span forward* task, while controls on average were able to repeat 6.3 ± 1.2 digits. These small differences likely became statistically significant only because of the large sample size.

Between patients with cerebellar stroke and their matched controls, small numerical differences were seen on the items *go/no-go* (MD: − 0.7; − 1.1, − 0.3) and *affect* (MD: − 0.4; − 0.7, − 0.1). These differences were statistically significant (*p* values < 0.002; Fig. S3 and Table S8, supplementary material).

### Test–retest (intrarater) and interrater reliability

Because of differential G-CCAS-S performance of patients and controls (as shown above), test–retest (i.e., intrarater) and interrater reliability were calculated separately for the subgroups of patients and controls. This allowed us to assess how consistently the CCAS-S measures cognitive performance in healthy and diseased subjects, with the patient subgroups being of particular interest. A control group was included to check if learning effects become relevant in repeated measurements. Learning in the context of CCAS-S assessment might be impaired in patients. In the literature, there is a large body of evidence for impairments in cerebellar patients for different forms of learning [32–36].

Test–retest (intrarater) reliability was assessed in 21 patients (range: 13–44 days; mean ± standard deviation: 24.4 ± 9.8 days) and 22 controls (14–35; 24.7 ± 6.5 days). Interrater reliability was assessed in 19 patients (15–47; 29.1 ± 10.2 days) and 23 controls (5–48; 25.3 ± 11.3 days). The *uncorrected* number of failed test items and the *uncorrected* total sum raw score in patients and controls at T1 and T2 are displayed in Table S9, supplementary material.

Considering the *uncorrected* number of failed test items, test–retest (intrarater) reliability was moderate in patients (ICC: 0.77) and good in controls (ICC: 0.88). The SEM (i.e., the inherent error of the G-CCAS-S) was determined to be 0.9 failed test items for patients and 0.5 for controls. This yielded a minimal detectable change at the 95% confidence interval (MDC95%) of 2.4 failed test items for patients and a MDC95% of 1.4 failed test items for controls. Test–retest (intrarater) reliability was improved considering the *uncorrected* total sum raw score both in patients (ICC: 0.89) and controls (0.91). These ICCs yielded an SEM of 4.1 and an MDC95% of 11.4 raw score points in patients and an SEM of 3.3 and an MDC95% of 9.2 raw score points in controls (Table 3).

**Table 3:** Test–retest (intrarater) and interrater reliability

Measure	Patients		Controls	
Test–retest (intrarater) reliability	Failed test items	Total sum score	Failed test items	Total sum score
ICC	0.77	0.89	0.88	0.91
SEM	0.9	4.1	0.5	3.3
MDC80%	2.2	10.5	1.3	8.5
MDC95%	2.4	11.4	1.4	9.2

Measure	Patients		Controls	
Interrater reliability	Failed test items	Total sum score	Failed test items	Total sum score
ICC	0.88	0.95	0.59	0.86
SEM	0.8	3.4	0.9	3.6
MDC80%	2.1	8.8	2.3	9.3
MDC95%	2.2	9.5	2.5	10.1

*ICC* intraclass correlation coefficient, *SEM* standard error of measure, *MDC80%/MDC95%* minimal detectable change at 80% or 95% confidence interval

Considering the *uncorrected* number of failed test items, interrater reliability was good (ICC: 0.88) in patients and moderate in controls (ICC: 0.59). The SEM was determined to be 0.8 failed test items in patients and 0.9 in controls. The MDC95% was 2.2 failed test items in patients and 2.5 in controls. Interrater reliability was again improved when considering the *uncorrected* total sum raw score both in patients (ICC: 0.95) and in controls (ICC: 0.86). These ICCs yielded an SEM of 3.4 in patients and 3.6 in controls and an MDC95% of 9.5 raw score points in patients and 10.1 in controls (Table 3).

### Internal consistency

The interrelatedness of the single test items, i.e., Cronbach’s alpha, was calculated as an additional measure of reliability. Note that for calculation of Cronbach’s alpha, only the *uncorrected* single item raw scores were used. Pass/fail criterion was not used, because Cronbach’s alpha cannot be applied to nominal data such as the pass/fail criterion on single test items [37]. Internal consistency is typically calculated for the whole group of patients and controls. This approach ensures that reliability is assessed across different populations, providing a more comprehensive understanding of its consistency [31]. Cronbach’s alpha yielded a value of 0.71. The effect on Cronbach’s alpha if single items are removed from the scale is shown in Table 4.

**Table 4:** Interrelatedness of test items

G-CCAS-S in its present form	Cronbach’s alpha
	0.71
**Test** **item**	**Cronbach’s alpha if test is removed**
Semantic fluency	0.64
Phonematic fluency	0.66
Category switching	0.65
Digit span forward	0.71
Digit span backward	0.70
Cube drawing	0.69
Verbal recall	0.71
Similarities	0.69
Go/no-go	0.71
Affect	0.70

Note, that the removal of the word fluency tests (*semantic fluency*, *phonematic fluency*, *category switching*) would worsen Cronbach’s alpha, while the removal of other items would not change Cronbach’s alpha substantially.

Finally, possible learning effects were assessed by correlating the time interval between baseline (T1) and retest (T2) with the change (Δ) of *uncorrected* failed test items or *uncorrected* total sum raw score between T1 and T2. All correlations were calculated for the subgroups separately and for all patients respective controls pooled together. All correlations were not significant (*p* values ≥ 0.185, Spearman’s rank correlation coefficient; Table S10, supplementary material), indicating that no substantial learning effects were present.

## Discussion

This manuscript presents the results of the German CCAS-S validation study. Moreover, for the first time, correction formulas were introduced which account for the known age, education, and sex effects on CCAS-S performance [9, 14, 16]. The correction formulas are easily applicable in clinical routine assessments, since no adaptations to the published and already used G-CCAS-S [8] are necessary. The correction formulas significantly improved diagnostic accuracy of the G-CCAS-S, particularly for the total sum raw score. Therefore, we recommend using the *corrected* total sum raw score for evaluation instead of the *uncorrected* number of failed items, as proposed originally by Hoche and colleagues [7].

We were able to replicate former results of our [13, 14] and other groups [9, 16] of a high rate of false-positive results in healthy participants partly grounded in age and education, and to lesser degree in sex effects. We also demonstrated that application of the newly developed correction formulas decreased the number of false-positive results in controls, especially when considering the total sum raw score. The rate of false positives was 13% when using the total sum raw score at the 95% prediction interval. For comparison, using the correction formula for failed test items, the false-positive rate was 40%, and using the method of Hoche et al. [7], it was 67%. In the original US–American validation trial, the false-positive rate was 26% for the exploratory and 7% for the validation cohort, respectively [7] which is much lower than our results using the correction formula for the number of failed test items. A high rate of false positives has also been reported by Rodríguez-Labrada et al. (94%) [9] and Selvadurai et al. (54%) [16]. It is important to note that the controls in the study of Hoche et al. were significantly younger (mean age: ~ 40 yrs) [7] than those in our cohort (~ 53–56 yrs), and in the study of Rodríguez-Labrada et al. (~ 48 yrs; *n* = 64 controls) [9]. The control cohort of Selvadurai and colleagues was also comparatively young (~ 40 yrs), but the sample size was much smaller (*n* = 37).

Besides the false-positive rate, in the current study, the correct positive rate in patients was reduced when using the correction formulas (failed items: 69%; total sum raw score: 48%) compared to the method of Hoche et al. (failed items: 87%). However, since the method of Hoche et al. does not account for the mentioned demographic effects, it is likely that the presence of CCAS is overestimated using that approach. In other words, if not accounting for age, education, and sex effects, these demographic effects could mistakenly be classified as *abnormal* findings, both in patients and in controls. In contrast, when using the newly developed correction formula for the total sum raw score with only 13% false-positive results, 48% of all patients were correctly identified as patients. Although the exact prevalence of CCAS in cerebellar disorders is unknown and no gold standard for diagnosing CCAS exists, it is likely that not all patients suffer from CCAS. According to the functional topography of the cerebellum, the localization of focal cerebellar lesions or the pattern of cerebellar atrophy determine if motor, non-motor, or both syndromes occur [38]. Three non-motor representations have been described in the cortex of the posterolateral cerebellar hemispheres and two main motor representations are known to be located in the anterior with some extension into the posterior cerebellar lobe [39]. Likewise, the dentate nucleus consists of a non-motor and a motor area [37, 40]. The finding of CCAS in some, but not all cerebellar patients has been reported for different entities. For example, in a sample of cerebellar stroke patients, cognitive deficits were detected in ~ 60% within the subacute and ~ 50% in the chronic phase [41]. Other studies that have applied the CCAS-S to patients with cerebellar stroke have reported 3 or more failed items in 21/25 (~ 84%) patients during the acute [19] and in 12/22 (~ 55%) patients in the chronic phase [20]. Overall, it seems that patients in the (sub)acute phase perform worse than those in the chronic stage. In accordance with this hypothesis, one study which used a detailed neuropsychological assessment found some degree of cognitive recovery over the observation of 4 months following cerebellar stroke [42]. This finding is also consistent with the original paper by Schmahmann and Sherman [5] describing CCAS which demonstrated cognitive recovery at follow-up visits. The majority (18/21) of the patients with cerebellar stroke in our study were in the chronic stage (> 6 months after cerebellar stroke). A *corrected abnormal* number of failed test items was detected in 56%, a *corrected abnormal* total sum score in 24%, and an *abnormal* test considering the *uncorrected* method of Hoche et al. [7] in 76% of our stroke patients.

Another recent study found that ~ 19–37% (failing none or one CCAS-S item) of 309 patients with SCAs performed within the range of healthy controls [16]. Despite the fact that the CCAS may not be present in every patient, the CCAS-S in its current version is likely too insensitive to detect subtle forms of CCAS. Indeed, results from a recent cluster analysis of the current data set from our group indicate that even in patients which perform within the same range as controls on the CCAS-S, some cognitive domains are impaired [43].

The finding that the age-, education-, and sex-corrected total sum raw score is more selective compared to the *corrected* number of failed test items may be explained by the relative greater weighting of the word fluency tests: while *semantic* (maximum score: 26 raw score points) and *phonematic word fluency* (19 points) as well as the *category switching* test item (15 points) add up to 50% of the total sum raw score (range: 0–120), the pass/fail criterion for the three word fluency test items adds up to a maximum of three failed items representing 30% of the total of failed test items (range: 0–10). Moreover, the range is wider for the total sum raw score which makes the separation between patients and controls finer graded.

Also, the finding that females have a lower risk ratio (0.88) to fail a test item and that they on average score ~ 2.8 raw score points higher than males likely is driven by the word fluency items: women scored significantly higher raw scores on the *phonematic word fluency* and the *category switching* test item, while men scored higher only on the *cube drawing* test item. Thus, the word fluency tests are again overrepresented compared to the *cube drawing* test item being the only item that captures visuospatial cognitive functions. Better performance of females on *phonematic word fluency* tests has also been described by others [44–48]. For *semantic word fluency*, mixed results have been published [49]. One recent study hypothesized that sex differences on semantic fluency tests might depend on the sematic category. For example, females might be better to name vegetables, because they tend to purchase the groceries. Contrary, men might be better to name tools, because they use them more frequently [50]. Better performance of men in visuospatial cognitive tasks has also been reported [44].

The necessity of correction formulas becomes even more obvious when comparing subgroups of patients which differ regarding sex distribution, age, and educational level. As yet, studies that have compared cognitive performance in different cerebellar diseases [15, 17, 19, 20] including a large recent study in patients with different spinocerebellar ataxias [16] and a preliminary evaluation from our group [13] have neglected the different age range in distinct cerebellar disorders. For example, FRDA usually has an onset before the age of 20 years [51]. In contrast, spinocerebellar ataxias occur in adulthood: SCA14 (~ 30 yrs), SCA3 (~ 40 yrs), and SCA6 (~ 50 yrs) [52]. Also, cerebellar strokes usually occur in later adulthood (~ 65 yrs) [53]. In our sample, FRDA patients were on average 39, SCA14 patients 56, SCA3 patients 53, PICA stroke patients 58, and SCA6 patients 62 years old.

According to the method of Hoche et al., FRDA, SCA14, SCA3, PICA stroke, and SCA6 patients would have performed similarly when tested with the CCAS-S: 77% of the FRDA, 89% of the SCA14, 87% of the SCA3, 77% of the PICA stroke, and 89% of the SCA6 patients had an *uncorrected abnormal* test result by that method to some degree. In contrast, when correcting for age, education, and sex effects, FRDA patients performed notably better than SCA14, SCA3, PICA stroke, and SCA6 patients (*abnormal* tests: FRDA: 45%, SCA14: 58%, SCA3: 64%, PICA stroke: 67%, SCA6: 72%). The rate of correct positives was clearly lower when considering the *corrected* total sum raw score which has a more conservative threshold to maximize selectivity (that is reducing the rate of false-positive results in healthy controls): FRDA: 23%, SCA14: 42%, SCA3: 33%, PICA stroke: 22%, SCA6: 17%. The difference between the subgroups when comparing *corrected* test results in contrast to very similar values for the *uncorrected* test results according to the method of Hoche et al. [7] underscores the importance of *corrected* test results when comparing different study populations.

Some of the test items appear to better differentiate between patients and controls than others. We were able to confirm results from a preliminary analysis of our group [13], showing that the word fluency tasks differentiated best between patients and controls. This observation has also been made by other groups [9, 15, 16]. In contrast, controls failed similarly often on the digit span tasks as patients. Preserved digit span in cerebellar disease has been reported by others as well [15, 54–56]. These findings also agree with a meta-analysis that included ten studies examining CCAS in a total of 212 patients with isolated cerebellar lesions. The meta-analysis showed that patients performed significantly worse on word fluency tests, but not on other tests which are also part of the CCAS-S (*go/no-go* and *digit span backward*). Moreover, the meta-analysis found that the *Stroop-Test* was the test which had shown the largest effect size and therefore would have been the first-choice test to detect executive deficits in cerebellar patients [3]. This test is currently not part of the CCAS-S. Other promising tests of neuropsychiatric functions, which are not yet part of the CCAS-S are tests of social cognition. Social cognition relies on the cerebellum and is required to understand, generate, and regulate social behavior [57–61]. One test of social cognition is the *Picture Sequencing Test* which requires the participant to arrange cards depicting (social) actions of persons in a meaningful sequence [12]. This computer-based test is less prone to rater-based biases compared to the CCAS-S which might improve the SEM and the MDC. Other tests that assess social cognition would be the *Emotion Attribution Test* [62], the *Faux Pas Test* [54], or the *Reading the Mind in the Eyes Test* [63]. All these tests have been reported to sensitively detect impairments of social cognition in cerebellar patients or patients with neurodevelopmental disorders where the cerebellum is discussed to play a role, like autism-spectrum disorders [54, 57, 62, 63].

In contrast to the original US-American validation trial [7], the current study assessed test–retest (intrarater) and interrater reliability of the CCAS-S. Test–retest and interrater reliability was good to excellent (ICC: 0.77–0.95), except for the number of failed test items in the subgroup of controls which received the interrater testing (ICC: 0.59). These ICCs are within the same range as reported in the validation study of the Spanish CCAS-S [9]. In all subgroups, the intraclass correlation coefficient (ICC) was higher for the total sum raw score than for the number of failed test items. The standard error of measurement (SEM), that is the inherent measuring error of the CCAS-S, ranged between 0.8 and 0.9 failed items or 3.4 and 4.1 raw score points in patients. These SEMs yielded a minimal detectable change at the 95% confidence interval (MDC95%) of 2.2–2.4 failed test items or 9.5–11.4 raw score points. Practically, these values would mean that a real change of test performance can first be safely assumed if patients fail two additional or less test items or if their total sum raw score changes by ~ 9–11 raw score points on the second test session. Since these values are quite large, the ability of the CCAS-S to be useful for follow-up examinations and particularly in treatment studies is limited. However, the current data support the recommendation of the authors of the original US-American CCAS-S [7] that if the scale is used for follow-up examinations, the total sum raw score should be used for comparison instead of the number of failed test items [7].

Internal consistency of the G-CCAS-S, expressed by Cronbach’s alpha which was 0.71, is considered “acceptable” according to the previous literature [7, 31]. This result is similar to the internal consistency of the Spanish (α = 0.74) [9] and the Portuguese adaptation of the CCAS-S (α = 0.75) [10], but it is higher than in the original US-American version of the CCAS-S (α = 0.59) [7]. Like in the current study, Rodríguez-Labrada and colleagues have reported that internal consistency decreases if the three word fluency tests are removed from the scale [9], whereas removal of all other test items in the current study and all test items except the *cube drawing* test items in the study of Rodríguez-Labrada did not change Cronbach’s alpha substantially. This indicates again that the word fluency test items are essential to the CCAS-S, whereas other test items may be redundant.

Although the introduced correction formulas improve diagnostic accuracy of the German version of the CCAS-S, it remains unclear if the correction formulas are completely applicable to versions of the CCAS-S in other languages. While the difficulty of most test items should be the same in different languages (e.g., *cube drawing*, *digit span forward*, *semantic fluency*), the difficulty of the *phonematic fluency* tests might differ, because words that start with a certain letter likely have different frequencies in different languages. Another aspect that might impact test performance is that educational levels might differ between countries given different educational systems.

## Conclusions

This study showed that the German CCAS-S is a valid test to detect cognitive dysfunction in patients with cerebellar disorders on the group level. To improve the diagnostic accuracy of G-CCAS-S for individual evaluation without altering the existing scale, correction formulas were introduced which account for age, education, and sex effects. The corrections effectively reduced false-positive rates in healthy controls, especially when considering the total sum raw score which should be used as the preferred evaluation measure. However, the fact that sensitivity was only low to moderate and that some test items do not differentiate well between patients and controls, underscores the need for more sensitive (and at the same time) selective CCAS assessment instruments. The finding of a large minimal detectable change in follow-up examinations also supports this conclusion. Possible changes over time are of particular interest in clinical trials to evaluate the efficacy of an intervention. The inclusion of more word fluency tests, which distinguished best between patients and controls, or other tests for executive cognitive functions like the *Stroop-Test* or tests of social cognition, could possibly improve diagnostic accuracy when assessing for CCAS.

## Supplementary Information

Below is the link to the electronic supplementary material.

Supplementary file1 (DOCX 1135 KB)

## Data Availability

Data underlying the statistics and the figures are available from the corresponding author upon request.
