# Supplementary material for: Optimizing selectivity of the Cerebellar Cognitive Affective Syndrome Scale by use of correction formulas, and validation of its German version
Source: J Neurol. 2025 Apr 17;272(5):343. doi: 10.1007/s00415-025-13083-3 (PMC12006234; doi:10.1007/s00415-025-13083-3)
Supplement: Supplementary file 1 — Supplementary file1 (DOCX 1135 KB) [file 415_2025_13083_MOESM1_ESM.docx]

**Optimizing selectivity of the Cerebellar Cognitive Affective Syndrome Scale by use of correction formulas, and validation of its German version**

Andreas Thieme^1^, Kerstin Rubarth^2^, Raquel van der Veen^1^, Johanna Müller^1^, Jennifer Faber^3,4^, Miriam Barkhoff^3^, Martina Minnerop^5,6,7^, Saskia Elben^5,6^, Dana Huvermann^1,8^, Friedrich Erdlenbruch^1^, Adam M. Berlijn^7,8^, Patricia Sulzer^9,10^, Kathrin Reetz^11,12^, Imis Dogan^11,12^, Heike Jacobi^13^, Julia-Elisabeth Aktories^13^, Giorgi Batsikadze^1^, Qi Liu^1^, Benedikt Frank^1^, Martin Köhrmann^1^, Elke Wondzinski^14^, Mario Siebler^14^, Jürgen Konczak^15^, Matthis Synofzik^9,10^, Thomas Klockgether^3^, Frank Konietschke^2,16^, Sandra Roeske^3,*^, Dagmar Timmann^1,*^

**Table S1: Excluded participants.**

| **Exclusion criterion** | **Patients (n)** | **Controls (n)** |
| --- | --- | --- |
| Medical reasons  *(neurological disorder other than ataxia or primary psychiatric disorders in patients; brainstem involvement in cerebellar stroke patients; neurological/psychiatric disorders or intake of centrally acting drugs in controls)* | 25 | 23 |
| No native German speaker | 5 | 5 |
| Mistakes in test execution | 6 | 5 |

Abbreviations: n = number.

**Table S2: Patients’ characteristics.**

| **Patients with cerebellar degeneration (autosomal-dominant hereditary ataxias)** | | | | | | | |
| --- | --- | --- | --- | --- | --- | --- | --- |
| **Subject No.** | **Age**  **[yrs]** | **Education**  **[yrs]** | **Sex** | **Diagnosis** | **SARA**  **score** | **CCAS-S**  **failed  test items** | **CCAS-S**  **total  sum score** |
| Cer-deg-1 | 47 | 12 | f | SCA1 | 14.0 | 5 | 73 |
| Cer-deg-2 | 49 | 19 | m | SCA1 | 15.0 | 5 | 67 |
| Cer-deg-3 | 53 | 13 | m | SCA1 | 12.5 | 5 | 73 |
| Cer-deg-4 | 54 | 15 | m | SCA1 | 24.0 | 5 | 63 |
| Cer-deg-5 | 58 | 18 | m | SCA1 | 8.0 | 4 | 80 |
| Cer-deg-6 | 30 | 21 | f | SCA2 | 10.0 | 0 | 105 |
| Cer-deg-7 | 51 | 13 | m | SCA2 | 9.5 | 3 | 79 |
| Cer-deg-8 | 53 | 16 | f | SCA2 | 17.0 | 1 | 94 |
| Cer-deg-9 | 55 | 14 | m | SCA2 | 12.5 | 6 | 65 |
| Cer-deg-10 | 57 | 18 | f | SCA2 | 13.5 | 4 | 83 |
| Cer-deg-11 | 57 | 16 | f | SCA2 | 23.0 | 1 | 98 |
| Cer-deg-12 | 63 | 12 | m | SCA2 | *missing* | 6 | 58 |
| Cer-deg-13 | 22 | 12 | f | SCA3 | 0.0 | 2 | 96 |
| Cer-deg-14 | 23 | 15 | f | SCA3 | 0.0 | 1 | 103 |
| Cer-deg-15 | 23 | 15 | m | SCA3 | 2.5 | 1 | 108 |
| Cer-deg-16 | 31 | 11 | f | SCA3 | 18.5 | 4 | 77 |
| Cer-deg-17 | 35 | 14 | m | SCA3 | 12.0 | 2 | 91 |
| Cer-deg-18 | 36 | 13 | f | SCA3 | 8.0 | 3 | 87 |
| Cer-deg-19 | 39 | 17 | m | SCA3 | 9.0 | 3 | 80 |
| Cer-deg-20 | 40 | 15 | m | SCA3 | 5.5 | 1 | 99 |
| Cer-deg-21 | 40 | 16 | m | SCA3 | 22.5 | 4 | 69 |
| Cer-deg-22 | 41 | 17 | m | SCA3 | 15.0 | 3 | 82 |
| Cer-deg-23 | 42 | 14 | f | SCA3 | 3.5 | 1 | 104 |
| Cer-deg-24 | 43 | *missing* | f | SCA3 | 5,0 | 0 | 104 |
| Cer-deg-25 | 45 | 17 | f | SCA3 | 2.0 | 1 | 100 |
| Cer-deg-26 | 47 | 22 | m | SCA3 | 6.0 | 0 | 107 |
| Cer-deg-27 | 48 | 18 | m | SCA3 | 3.0 | 0 | 111 |
| Cer-deg-28 | 48 | 21 | m | SCA3 | 1.5 | 3 | 84 |
| Cer-deg-29 | 48 | 13 | f | SCA3 | 0.0 | 1 | 99 |
| Cer-deg-30 | 50 | 13 | f | SCA3 | 9.5 | 2 | 93 |
| Cer-deg-31 | 50 | 14 | m | SCA3 | 10.0 | 1 | 94 |
| Cer-deg-32 | 51 | 23 | m | SCA3 | 9.0 | 0 | 112 |
| Cer-deg-33 | 51 | 13 | f | SCA3 | 19.0 | 1 | 93 |
| Cer-deg-34 | 51 | 17 | f | SCA3 | 15.0 | 3 | 82 |
| Cer-deg-35 | 51 | 24 | m | SCA3 | 14.0 | 1 | 100 |
| Cer-deg-36 | 52 | 12 | f | SCA3 | 11.0 | 7 | 67 |
| Cer-deg-37 | 53 | 19 | m | SCA3 | 10.5 | 1 | 100 |
| Cer-deg-38 | 53 | 18 | m | SCA3 | 17.0 | 2 | 88 |
| Cer-deg-39 | 53 | 16 | f | SCA3 | 12.0 | 1 | 86 |
| Cer-deg-40 | 53 | 16 | f | SCA3 | 26.5 | 3 | 80 |
| Cer-deg-41 | 53 | 16 | f | SCA3 | 21.5 | 4 | 79 |
| Cer-deg-42 | 54 | 13 | f | SCA3 | 13.0 | 0 | 101 |
| Cer-deg-43 | 54 | 17 | f | SCA3 | 11.0 | 1 | 97 |
| Cer-deg-44 | 58 | 18 | f | SCA3 | 12.0 | 1 | 102 |
| Cer-deg-45 | 59 | 11 | m | SCA3 | 3.0 | 4 | 85 |
| Cer-deg-46 | 61 | 21 | f | SCA3 | 24.5 | 3 | 90 |
| Cer-deg-47 | 61 | 13 | f | SCA3 | 14.5 | 2 | 93 |
| Cer-deg-48 | 62 | 12 | f | SCA3 | 10.0 | 4 | 84 |
| Cer-deg-49 | 63 | 13 | m | SCA3 | 17.5 | 2 | 73 |
| Cer-deg-50 | 65 | 13 | f | SCA3 | 13.0 | 2 | 88 |
| Cer-deg-51 | 65 | 13 | f | SCA3 | 27.5 | 3 | 80 |
| Cer-deg-52 | 67 | 12 | f | SCA3 | 20.5 | 1 | 95 |
| Cer-deg-53 | 67 | 18 | f | SCA3 | 34.0 | 6 | 65 |
| Cer-deg-54 | 70 | 16 | f | SCA3 | 17.0 | 0 | 99 |
| Cer-deg-55 | 72 | 12 | m | SCA3 | 11.0 | 4 | 85 |
| Cer-deg-56 | 77 | 14 | f | SCA3 | 32.0 | 7 | 66 |
| Cer-deg-57 | 81 | 16 | m | SCA3 | 21.0 | 2 | 99 |
| Cer-deg-58 | 33 | 19 | m | SCA6 | 2.0 | 5 | 79 |
| Cer-deg-59 | 49 | 17 | m | SCA6 | 13.0 | 2 | 104 |
| Cer-deg-60 | 52 | 15 | m | SCA6 | 6.0 | 5 | 86 |
| Cer-deg-61 | 55 | 12 | m | SCA6 | 17.0 | 2 | 90 |
| Cer-deg-62 | 55 | 23 | m | SCA6 | 2.0 | 1 | 109 |
| Cer-deg-63 | 55 | 13 | f | SCA6 | 19.5 | 3 | 96 |
| Cer-deg-64 | 59 | 15 | m | SCA6 | 10.0 | 3 | 95 |
| Cer-deg-65 | 59 | 13 | m | SCA6 | 18.5 | 0 | 104 |
| Cer-deg-66 | 62 | 12 | f | SCA6 | 6.5 | 3 | 92 |
| Cer-deg-67 | 64 | 9 | m | SCA6 | 16.0 | 3 | 83 |
| Cer-deg-68 | 66 | 19 | m | SCA6 | 13.0 | 1 | 97 |
| Cer-deg-69 | 66 | 15 | m | SCA6 | 14.5 | 1 | 94 |
| Cer-deg-70 | 67 | 13 | f | SCA6 | 7.5 | 0 | 115 |
| Cer-deg-71 | 74 | 12 | f | SCA6 | 20.0 | 3 | 72 |
| Cer-deg-72 | 77 | 11 | f | SCA6 | 15.5 | 10 | 54 |
| Cer-deg-73 | 78 | 13 | m | SCA6 | *missing* | 3 | 84 |
| Cer-deg-74 | 78 | 18 | m | SCA6 | 11.0 | 1 | 91 |
| Cer-deg-75 | 79 | 18 | m | SCA6 | 5.5 | 3 | 85 |
| Cer-deg-76 | 59 | 13 | m | SCA8 | 11.0 | 4 | 69 |
| Cer-deg-77 | 61 | 9 | m | SCA8 | 26.5 | 8 | 64 |
| Cer-deg-78 | 62 | 12 | f | SCA8 | 11.5 | 6 | 59 |
| Cer-deg-79 | 33 | 17 | f | SCA13 | 6.0 | 5 | 82 |
| Cer-deg-80 | 70 | 9 | m | SCA13 | 13.0 | 9 | 55 |
| Cer-deg-81 | 38 | 12 | f | SCA14 | 4.5 | 0 | 107 |
| Cer-deg-82 | 40 | 14 | m | SCA14 | 8.0 | 0 | 109 |
| Cer-deg-83 | 53 | 16 | f | SCA14 | 15.0 | 1 | 108 |
| Cer-deg-84 | 54 | 16 | m | SCA14 | 12.0 | 5 | 82 |
| Cer-deg-85 | 56 | 23 | f | SCA14 | 9.0 | 1 | 105 |
| Cer-deg-86 | 58 | 13 | m | SCA14 | 13.5 | 3 | 79 |
| Cer-deg-87 | 59 | 14 | f | SCA14 | 11.0 | 2 | 91 |
| Cer-deg-88 | 61 | 14 | m | SCA14 | 15.0 | 2 | 80 |
| Cer-deg-89 | 64 | 23 | m | SCA14 | 10.0 | 0 | 102 |
| Cer-deg-90 | 65 | 12 | f | SCA14 | 15.0 | 4 | 92 |
| Cer-deg-91 | 71 | 14 | m | SCA14 | 14.0 | 3 | 74 |
| Cer-deg-92 | 72 | 13 | f | SCA14 | 9.5 | 3 | 81 |
| Cer-deg-93 | 28 | 21 | m | SCA27B | 7.5 | 2 | 87 |
| Cer-deg-94 | 37 | 18 | m | SCA27B | 4.0 | 2 | 93 |
| Cer-deg-95 | 61 | 12 | f | SCA27B | 6.0 | 0 | 105 |
| Cer-deg-96 | 62 | 15 | m | SCA27B | 8.0 | 3 | 90 |
| Cer-deg-97 | 76 | 12 | f | SCA27B | 10.5 | 2 | 98 |
| Cer-deg-98 | 76 | 12 | f | SCA27B | 3.0 | 3 | 79 |
| Cer-deg-99 | 77 | 12 | m | SCA27B | 10.0 | 6 | 57 |
| Cer-deg-100 | 79 | 11 | f | SCA27B | 10.0 | 5 | 75 |
| Cer-deg-101 | 58 | 14 | m | SCA28 | 14.5 | 5 | 75 |
| Cer-deg-102 | 55 | 14 | m | SCA35 | 17.0 | 0 | 100 |
| Cer-deg-103 | 69 | 14 | m | SCA48 | 20.0 | 5 | 77 |
| Cer-deg-104 | 31 | 16 | f | LBSL | 20.5 | 6 | 75 |
| Cer-deg-105 | 22 | 15 | f | EA2 | 3.0 | 2 | 95 |
| Cer-deg-106 | 39 | 19 | m | EA2 | 2.0 | 2 | 90 |
| Cer-deg-107 | 53 | 14 | f | EA1 | 6.5 | 3 | 81 |
| Cer-deg-108 | 56 | 13 | f | EA | 12.0 | 1 | 95 |
| Cer-deg-109 | 56 | 8 | m | EA | 1.0 | 2 | 91 |
| Cer-deg-110 | 58 | 15 | f | EA | 9.5 | 1 | 99 |
| Cer-deg-111 | 59 | 14 | m | EA2 | 4.5 | 2 | 82 |
| Cer-deg-112 | 33 | 12 | m | ADCA3 | 3.0 | 5 | 79 |
| Cer-deg-113 | 50 | 20 | m | ADCA3 | 3.0 | 0 | 97 |
| Cer-deg-114 | 56 | 18 | f | ADCA3 | 10.0 | 4 | 81 |
| Cer-deg-115 | 63 | 13 | f | ADCA3 | 6.0 | 1 | 102 |
| Cer-deg-116 | 65 | 12 | f | ADCA3 | 12.0 | 3 | 85 |
| Cer-deg-117 | 61 | 14 | f | ADCA3 | 13.5 | 7 | 66 |
| Cer-deg-118 | 63 | 17 | m | ADCA3 | 15.0 | 6 | 65 |
| Cer-deg-119 | 68 | 14 | m | ADCA3 | 15.0 | 8 | 65 |
| Cer-deg-120 | 33 | 10 | m | ADCA1/2 | 15.0 | 7 | 70 |
| Cer-deg-121 | 47 | 10 | m | ARCA (ATP1A3 mutation) | 15.5 | 9 | 49 |
| Cer-deg-122 | 48 | 13 | f | ADCA1/2 | 8.0 | 1 | 111 |
| Cer-deg-123 | 51 | 16 | m | ADCA1/2 | 17.0 | 1 | 99 |
| Cer-deg-124 | 54 | 11 | f | ADCA1/2 | 14.0 | 7 | 72 |
| Cer-deg-125 | 59 | 13 | f | ADCA1/2 | 6.0 | 2 | 84 |
| Cer-deg-126 | 59 | 15 | f | ADCA1/2 | 7.0 | 2 | 79 |
| Cer-deg-127 | 59 | 10 | f | ADCA1/2 | 13,0 | 9 | 43 |
| Cer-deg-128 | 65 | 15 | m | ADCA1/2 | 11.5 | 4 | 73 |
| Cer-deg-129 | 66 | 12 | m | ADCA1/2 | 22.5 | 4 | 88 |
| Cer-deg-130 | 68 | 11 | m | ADCA1/2 | 11.0 | 3 | 75 |
| Cer-deg-131 | 68 | 11 | f | ADCA1/2 | 14.5 | 7 | 73 |
| Cer-deg-132 | 75 | 12 | m | ADCA1/2 | 4.0 | 6 | 68 |
| **Patients with cerebellar degeneration (autosomal-recessive hereditary ataxias)** | | | | | | | |
| **Subject No.** | **Age**  **[yrs]** | **Education**  **[yrs]** | **Sex** | **Diagnosis** | **SARA**  **score** | **CCAS-S**  **failed**  **test items** | **CCAS-S**  **total**  **sum score** |
| Cer-deg-133 | 28 | 14 | m | SCAR8 (SYNE) | 4.0 | 2 | 90 |
| Cer-deg-134 | 29 | 14 | m | SCAR8 (SYNE) | 4.5 | 2 | 89 |
| Cer-deg-135 | 54 | 9 | f | SCAR8 (SYNE) | 18.0 | 5 | 78 |
| Cer-deg-136 | 20 | 13 | m | SCAR9 (FXN) | 28.0 | 1 | 101 |
| Cer-deg-137 | 21 | 13 | m | SCAR9 (FXN) | 9.0 | 2 | 99 |
| Cer-deg-138 | 24 | 13 | m | SCAR9 (FXN) | 16.0 | 0 | 100 |
| Cer-deg-139 | 25 | 19 | m | SCAR9 (FXN) | 29.5 | 1 | 106 |
| Cer-deg-140 | 27 | 19 | f | SCAR9 (FXN) | 18.5 | 1 | 94 |
| Cer-deg-141 | 28 | 17 | f | SCAR9 (FXN) | 33.0 | 3 | 92 |
| Cer-deg-142 | 28 | 16 | f | SCAR9 (FXN) | 21.0 | 1 | 98 |
| Cer-deg-143 | 29 | 19 | m | SCAR9 (FXN) | 13.5 | 1 | 99 |
| Cer-deg-144 | 31 | 20 | m | SCAR9 (FXN) | 10.5 | 3 | 97 |
| Cer-deg-145 | 31 | 16 | m | SCAR9 (FXN) | 7.0 | 0 | 114 |
| Cer-deg-146 | 31 | 7,0 | m | SCAR9 (FXN) | 31,0 | 5 | 65 |
| Cer-deg-147 | 35 | 16 | m | SCAR9 (FXN) | 17.5 | 3 | 85 |
| Cer-deg-148 | 37 | 10 | m | SCAR9 (FXN) | *missing* | 4 | 80 |
| Cer-deg-149 | 38 | 18 | f | SCAR9 (FXN) | 12.0 | 1 | 91 |
| Cer-deg-150 | 49 | 16 | f | SCAR9 (FXN) | 14.0 | 0 | 106 |
| Cer-deg-151 | 52 | 15 | f | SCAR9 (FXN) | 16.0 | 1 | 95 |
| Cer-deg-152 | 54 | 17 | f | SCAR9 (FXN) | 22.0 | 0 | 100 |
| Cer-deg-153 | 56 | 20 | f | SCAR9 (FXN) | 27.5 | 2 | 108 |
| Cer-deg-154 | 56 | 13 | f | SCAR9 (FXN) | 29.0 | 2 | 94 |
| Cer-deg-155 | 57 | 20 | m | SCAR9 (FXN) | 22.5 | 0 | 101 |
| Cer-deg-156 | 62 | 18 | m | SCAR9 (FXN) | 24.5 | 2 | 90 |
| Cer-deg-157 | 75 | 12 | f | SCAR9 (FXN) | 29.0 | 4 | 78 |
| Cer-deg-158 | 32 | 15 | f | SCAR10 (ANO10) | 8.0 | 4 | 85 |
| Cer-deg-159 | 32 | 13 | f | SCAR10 (ANO10) | 12.0 | 0 | 107 |
| Cer-deg-160 | 53 | 14 | f | SCAR10 (ANO10) | 19.5 | 4 | 88 |
| Cer-deg-161 | 27 | 17 | m | SCAR 12 ( POLG) | 13.0 | 1 | 86 |
| Cer-deg-162 | 51 | 14 | f | SCAR12 (POLG) | 26.0 | 4 | 67 |
| Cer-deg-163 | 23 | 18 | m | SCAR15 (SETX) | 11.0 | 3 | 85 |
| Cer-deg-164 | 53 | 13 | m | SCAR16 (STUB1) | 12.5 | 3 | 77 |
| Cer-deg-165 | 32 | 15 | m | SCAR18 (SACS) | 24.0 | 1 | 92 |
| Cer-deg-166 | 49 | 20 | m | SCAR23 (PNPLA6) | 14.0 | 4 | 84 |
| Cer-deg-167 | 61 | 12 | f | SCAR25 (RFC1) | 5.5 | 1 | 89 |
| Cer-deg-168 | 62 | 12 | m | SCAR25 (RFC1) | 26.5 | 5 | 69 |
| Cer-deg-169 | 71 | 18 | m | SCAR25 (RFC1) | 20.0 | 0 | 106 |
| Cer-deg-170 | 47 | 11 | m | SCAR36 (SPG7) | 15,0 | 6 | 76 |
| **Patients with cerebellar degeneration (sporadic ataxias)** | | | | | | | |
| **Subject No.** | **Age**  **[yrs]** | **Education**  **[yrs]** | **Sex** | **Diagnosis** | **SARA**  **score** | **CCAS-S**  **failed**  **test items** | **CCAS-S**  **total sum score** |
| Cer-deg-171 | 53 | 16 | m | MSA-C | 10.5 | 4 | 83 |
| Cer-deg-172 | 62 | 17 | m | MSA-C | 17.5 | 2 | 83 |
| Cer-deg-173 | 62 | 14 | f | MSA-C | 28.5 | 3 | 75 |
| Cer-deg-174 | 64 | 13 | f | MSA-C | 14.5 | 1 | 90 |
| Cer-deg-175 | 64 | 17 | m | MSA-C | 23.0 | 9 | 48 |
| Cer-deg-176 | 66 | 16 | m | MSA-C | 22.5 | 4 | 58 |
| Cer-deg-177 | 53 | 13 | f | SAOA | 12.5 | 3 | 90 |
| Cer-deg-178 | 57 | 18 | m | SAOA | 7.0 | 2 | 99 |
| Cer-deg-179 | 59 | 13 | m | SAOA | 28.5 | 2 | 101 |
| Cer-deg-180 | 65 | 13 | f | SAOA | 11.5 | 1 | 97 |
| Cer-deg-181 | 65 | 13 | m | SAOA | 11.0 | 3 | 97 |
| Cer-deg-182 | 70 | 17 | m | SAOA | 14.0 | 4 | 85 |
| Cer-deg-183 | 74 | 8 | m | SAOA | 9.0 | 2 | 85 |
| Cer-deg-184 | 79 | 12 | f | SAOA | 9.0 | 9 | 47 |
| Cer-deg-185 | 80 | 13 | f | SAOA | 9.0 | 2 | 82 |
| Cer-deg-186 | 35 | 21 | m | Autoimmune ataxia | 10.0 | 2 | 97 |
| Cer-deg-187 | 59 | 16 | m | Autoimmune ataxia | 23.5 | 4 | 80 |
| Cer-deg-188 | 75 | 18 | m | Autoimmune ataxia | 14.5 | 2 | 79 |
| **Patients with cerebellar stroke** | | | | | | | |
| **Subject No.** | **Age**  **[yrs]** | **Education**  **[yrs]** | **Sex** | **Diagnosis** | **SARA**  **score** | **CCAS-S**  **failed**  **test items** | **CCAS-S**  **total**  **sum score** |
| Cer-str-1 | 30 | 18 | m | PICA right | 0.0 | 2 | 95 |
| Cer-str-2 | 47 | 16 | m | PICA right | 0.0 | 0 | 107 |
| Cer-str-3 | 50 | 21 | m | PICA right | 0.0 | 0 | 108 |
| Cer-str-4 | 57 | 12 | f | PICA right | 1.5 | 7 | 82 |
| Cer-str-5 | 63 | 14 | m | PICA right | 3.0 | 2 | 89 |
| Cer-str-6 | 68 | 19 | m | PICA right | 0.5 | 0 | 105 |
| Cer-str-7 | 68 | 8 | f | PICA right | 3.0 | 3 | 71 |
| Cer-str-8 | 84 | 11 | f | PICA right | 5.5 | 6 | 75 |
| Cer-str-9 | 19 | 13 | m | PICA left | 0.0 | 2 | 94 |
| Cer-str-10 | 48 | 11 | f | PICA left | 1.0 | 3 | 94 |
| Cer-str-11 | 51 | 16 | f | PICA left | 1.0 | 0 | 104 |
| Cer-str-12 | 56 | 16 | m | PICA left | 0.0 | 1 | 96 |
| Cer-str-13 | 59 | 17 | m | PICA left | 1.0 | 1 | 87 |
| Cer-str-14 | 59 | 18 | m | PICA left | 2.0 | 2 | 100 |
| Cer-str-15 | 64 | 18 | m | PICA left | 6.0 | 2 | 102 |
| Cer-str-16 | 65 | 18 | m | PICA left | 3.5 | 2 | 91 |
| Cer-str-17 | 72 | 9 | m | PICA left | 0.0 | 2 | 84 |
| Cer-str-18 | 53 | 18 | m | SUCA right | 3.5 | 1 | 104 |
| Cer-str-19 | 69 | 12 | m | SUCA left | 4.0 | 0 | 109 |
| Cer-str-20 | 51 | 14 | f | PICA right &  PICA left | 5.0 | 5 | 72 |
| Cer-str-21 | 50 | 15 | m | PICA right &  SUCA left | 2.0 | 1 | 97 |

Abbreviations: Cer-deg = patients with cerebellar degeneration, Cer-str = patients with focal lesions due to isolated cerebellar stroke, SCA 1/ 2/ 3/ 6/ 8/ 13/ 14/ 27B/ 28/ 35/ 48 = spinocerebellar ataxia type 1/ 2/ 3/ 6/ 8/ 13/ 14/ 27B/ 28/ 35/ 48, LBSL = leukoencephalopathy with brainstem and spinal cord involvement and lactate elevation, EA (2) = episodic ataxia (type 2), ADCA 1/ 2/ 3 = autosomal-dominant ataxia type 1/ 2/ 3; SCAR 8/ 9/ 10/ 12/ 15/ 16/ 18/ 23/ 25/ 36 = spinocerebellar ataxia recessive type 8/ 9/ 10/ 12/ 15/ 16/ 18/ 23/ 25/ 36. Note, that for autosomal-recessive ataxias the gene abbreviation is provided in parentheses since some recessive ataxias are commonly referred to according to the name of the affected gene, e.g. , SYNE1-associated ataxia, POLG-associated ataxia, etc.: SYNE1 = synaptic nuclear envelope 1, FXN = frataxin, ANO10 = anoctamin 10, POLG = polymerase gamma, STEX = senataxin, STUB1 = STIB1 homology and U-box containing protein 1, SACS = spastic ataxia type Charlevoix-Saguenay, PNPLA6 = patatin-like phospholipase domain-containing protein 6, RFC1 = replication factor C subunit 1, SPG7 = spastic paraplegia 7; MSA-C = multisystem atrophy – cerebellar type, SAOA = sporadic adult onset ataxia of unknown etiology; PICA = stroke in the posterior inferior cerebellar artery territory, SUCA = stroke in the superior cerebellar artery territory.

**Table S3: CCAS-Scale items and assessment criteria.**

| **CCAS-Scale item** | **Instructions** | **Assessment criteria** | |
| --- | --- | --- | --- |
|  |  | **Raw Score**  **(minimum-maximum)** | **Fail score** |
| **Semantic fluency** | Please name as many animals or living creatures as you can in one minute. | 0-26  *one point per correct word* | ≤ 15 |
| **Phonematic fluency** | Please name as many words as you can in one minute that start with the letter F. Do not use names of people or places or repeat the same word in different forms. | 0-19  *one point per correct word* | ≤ 9 |
| **Category switching** | Please name a type of vegetable and then a type of profession or job, then another vegetable and another profession, and so on, switching between the two lists. Name as many as you can in one minute. | 0-15  *one point per correct alternation* | ≤ 9 |
| **Verbal recall** | I am going to read you a list of words which I would like you to learn. Please repeat these words. I am going to ask you to give them back in a few minutes.  (After the task *cube drawing*, the participant is asked to give the words back.) | 0-15  *three points per correct word if remembered spontaneously, two points if remembered with category cue, one point if remembered from multiple choice alternatives* | ≤ 10 |
| **Digit span forward** | I am going to read you some numbers. Please repeat them in exactly the same order. | 0-8  *one point per digit of longest correct string of numbers* | ≤ 5 |
| **Digit span backward** | Now please say these numbers backwards, in reverse order. | 0-6  *one point per digit of longest correct string of numbers* | ≤ 3 |
| **Cube drawing** | Please draw a cube – a six-sided box, make it transparent or see-through.  *(If task cannot be solved, the following task is required from the participant: Please copy the cube shown on page 2.)* | 0-15  *fifteen points if cube consists of twelve lines and is 3-dimensional; if cube does not consist of twelve lines or is not 3-dimensional, administer cube copying;*  *Cube copying: maximum score twelve points; one point per line,* subtract *one point if cube is not 3-dimensional and one point for each missing or additional line* | ≤ 11 |
| **Similarities** | How are the following words alike; what is the same about them? | 0-8  *two points per word pair for abstract answer, one point per word pair for concrete answer* | ≤ 6 |
| **Go/no-go** | I am going to tap the table. When I tap once, please raise your finger, then put it back down again. When I tap twice, do not do anything. | 0-2  *two points for no errors, one point for one error, zero points for two or more errors* | 0 |
| **Affect** | Rater assesses if the following are present, incorporating input from patient and/or caregiver:   - Difficulty with focusing attention or mental flexibility - Emotionally labile, incongruous emotions, appears hopeless or depressed - Shows easy sensory overload or avoidant behaviors - Expresses illogical thoughts or paranoia - Lacks empathy, is apathetic, or has blunted affect - Angry or aggressive, irritable, oppositional, difficulty with social cues and social boundaries | 0-6  *six points if none is present, subtract one point for each item present* | ≤ 4 |
| **Sum scores** | Sum scores are calculated by summation of the single test item scores. | **Total Sum Score**  0-120 | **Failed test items score**  0-10 |

Additional information: For further details and comprehensive test instructions, please refer to Thieme et al. (2020) [4], where the German CCAS-Scale was originally published, along with detailed test instructions.

**Table S4: Demographics of matched groups.**

|  | **Cer-deg** | **Con-deg** | **Cer-str** | **Con-str** |
| --- | --- | --- | --- | --- |
| **Age**  [mean ± SD (yrs)] | 53.0 ± 14.9 | 53.0 ± 15.6 | 56.3 ±14.2 | 56.0 ± 14.4 |
| **Level of education**  [mean ± SD (yrs)] | 15.0 ± 3.1 | 15.5 ± 3.0 | 14.9 ± 3.5 | 15.2 ±3.3 |
| **Sex distribution**  (n males / n females) | 97 / 79 | 84 / 92 | 15 / 6 | 15 / 6 |
| **Total n of participants** | 176 | 176 | 21 | 21 |

Abbreviations: Cer-deg = patients with cerebellar degeneration, Con-deg = matched controls for Cer-deg, Cer-str = patients with focal lesions due to isolated cerebellar stroke, Con-str = matched controls for Cer-str, SD = standard deviation, yrs = years, n = number.

**M1. Correlation analyses.**

Methods

To examine possible relationships between cognitive and motor symptoms in cerebellar patients, correlations between the performance on the G-CCAS-S (that is: the *uncorrected* number of failed test items respectively the *uncorrected* total sum raw score) and the SARA and ICARS score as well as the INAS count were examined. Moreover, possible relationships between the G-CCAS-S scores and age, years of education, sex, and the disease duration in years were analysed.

In controls, possible relationships between the *uncorrected* number of failed test items respectively the *uncorrected* total sum raw score and age, education, and sex were examined. Because data were not normally distributed, Spearman’s rank correlation coefficient was used for correlation analyses of continuous variables. Fisher’s exact test was used for categorial variables.

Results

In patients, the *uncorrected* total number of failed test items was positively correlated with age (r = 0.223, p < 0.001) and negatively correlated with the level of education (r = -0.366, p < 0.001). The *uncorrected* total sum raw score was positively correlated with the level of education (r = 0.377, p < 0.001) and inversely correlated with age (r = -0.294, p < 0.001). Both variables were also correlated with the SARA (failed items: r = 0.236, p < 0.001; sum raw score: r = -0.283, p < 0.001) and ICARS score (failed items: r = 0.348, p < 0.001; sum raw score: r = -0.355, p < 0.001), as well as the INAS count (failed items: r = 0.175, p = 0.022; sum raw score: r = -0.243, p = 0.001). However, no correlation was found between these cognitive variables and disease duration (p-values > 0.120; Tab. S5).

In healthy controls, the *uncorrected* total number of failed test items was positively correlated with age (r = 0.358, p < 0.001) and negatively correlated with the level of education (r = -0.295, p < 0.001). The *uncorrected* total sum raw score was positively correlated with level of education (r = 0.281, p < 0.001) and inversely correlated with age (r = -0.327, p < 0.001; Tab. S6).

**Table S5: Correlation analyses in patients.**

| **Measure** | **CCAS-S**  **total**  **sum score** | **SARA score** | **ICARS**  **score** | **INAS**  **count** | **Disease duration (yrs)** | **Age**  **(yrs)** | **Education**  **(yrs)** |
| --- | --- | --- | --- | --- | --- | --- | --- |
| **CCAS-S**  **failed**  **test items** | **r = -0.874**  **p < 0.001***  **n = 209** | **r = 0.236**  **p < 0.001***  **n = 206** | **r = 0.348**  **p < 0.001***  **n = 125** | **r = 0.175**  **p = 0.022***  **n = 170** | r = 0.080  p = 0.248  n = 208 | **r = 0.223**  **p < 0.001***  **n = 209** | **r = -0.366**  **p < 0.001***  **n = 208** |
| **CCAS-S**  **total**  **sum score** |  | **r = -0.283**  **p < 0.001***  **n = 206** | **r = -0.355**  **p < 0.001***  **n = 125** | **r = -0.243**  **p < 0.001***  **n = 170** | r = -0.107  p = 0.123  n = 208 | **r = -0.294**  **p < 0.001***  **n = 209** | **r = 0.377**  **p < 0.001***  **n = 208** |
| **SARA**  **Score** |  |  | **r = 0.959**  **p < 0.001***  **n = 125** | **r = 0.635**  **p < 0.001***  **n = 170** | **r = 0.418**  **p < 0.001***  **n = 205** | r = 0.135  p = 0.053  n = 206 | r = -0.015  p = 0.834  n = 205 |
| **ICARS score** |  |  |  | **r = 0.602**  **p < 0.001***  **n = 123** | **r = 0.356**  **p < 0.001***  **n = 124** | r = 0.066  p = 0.467  n = 125 | r = -0.068  p = 0.448  n = 125 |
| **INAS**  **Count** |  |  |  |  | **r = 0.305**  **p < 0.001***  **n = 169** | r = -0.043  p = 0.574  n = 170 | r = 0.037  p = 0.631  n = 170 |
| **Disease duration (yrs)** |  |  |  |  |  | r = 0.123  p = 0.077  n = 208 | r = -0.007  p = 0.922  n = 207 |
| **Age**  **(yrs)** |  |  |  |  |  |  | **r = -0.262**  **p < 0.001***  **n = 208** |

Abbreviations: CCAS-S = Cerebellar Cognitive Affective Syndrome Scale, SARA = Scale for the Assessment and Rating of Ataxia, ICARS = International Cooperative Ataxia Rating Scale, INAS = Inventory of Non-Ataxia Signs, yrs = years.

**Table S6: Correlation analyses in controls.**

| **Measure** | **CCAS-S**  **total**  **Sum score** | **Age**  **(yrs)** | **Education (yrs)** |
| --- | --- | --- | --- |
| **CCAS-S**  **failed**  **test items** | **r = -0.772**  **p < 0.001***  **n = 232** | **r = 0.358**  **p < 0.001***  **n = 232** | **r = -0.295**  **p < 0.001***  **n = 231** |
| **CCAS-S**  **total**  **sum score** |  | **r = -0.327**  **p < 0.001***  **n = 232** | **r = 0.281**  **p < 0.001***  **n = 231** |
| **Age**  **(yrs)** |  |  | r = -0.118  p = 0.072  n = 231 |

Abbreviations: CCAS-S = Cerebellar Cognitive Affective
Syndrome Scale, yrs = years.

**M2.** **Receiver operating curve analyses.**

Methods

To further explore the diagnostic accuracy of the G-CCAS-S, a receiver operating curve (ROC) analysis was performed in the matched groups graphing the true positive (sensitivity) versus the false positive rate (100% - selectivity) considering the *uncorrected* number of failed test items, the *uncorrected* total sum raw score and single test items’ raw scores. For all ROC analysis, the area under the curve (AUC) was calculated. An AUC of < 0.5 indicates that a test does not exceed chance level in discriminating patients from controls, while an AUC of 1 reflects a perfect relationship between the true positive and the false negative rate [5]. According to previous literature, an AUC between 0.5 and 0.7 is considered poor, between 0.7 and 0.8 is good, and > 0.8 is excellent [1-3].

Optimal cut-offs for *uncorrected* total failed test items and *uncorrected* total sum raw score were calculated using Youden’s Index (YI = sensitivity for a specific cut-off value + selectivity for that cut-off value - 1). YI indicates the cut-off for which the relationship between true positives (sensitivity) and false positives (100% - selectivity) is optimal [36, 46].

Results

ROC analysis revealed acceptable discriminative ability between patients with cerebellar degeneration and matched controls for the number of failed test items [AUC ± standard error (SE): 0.69 ± 0.03, p < 0.001, Fig. S1A], which was improved to good discriminative ability considering the *uncorrected* total sum raw score (AUC ± SE: 0.76 ± 0.03, p < 0.001, Fig. S1B). The optimal cut-off (Youden Index) to differentiate between patients with cerebellar degeneration and controls was three failed test items or 90 points considering the total sum raw score. These cut-off values showed good selectivity in controls (81% true negatives when using the cut-off of three failed test items and 83% when using the cut-off of 90 points on the total sum raw score). Sensitivity for these cut-offs (true positives), however, was poor to moderate (48% respectively 56%).

Comparing the group of patients with cerebellar stroke and controls delivered poor discriminative ability both for the *uncorrected* number of failed test items (AUC ± SE: 0.63 ± 0.09, p = 0.146, Fig. S1C) and for the *uncorrected* total sum raw score (AUC ± SE: 0.64 ± 0.09, p = 0.116, Fig. S1D). The optimal cut-off (Youden Index) to differentiate between patients with cerebellar stroke and controls was two failed test items or 97 points considering the total sum raw score. These cut-off values showed moderate selectivity in controls (71% true negatives when using the cut-off of two failed test items and 62% when using the cut-off of 97 points on the total sum raw score). Sensitivity for these cut-offs (true positives) was poor to moderate (57% respectively 62%).

**Figure S1: Receiver operating curves (ROC) for the number of *uncorrected* failed test items and the *uncorrected* total sum raw score.**


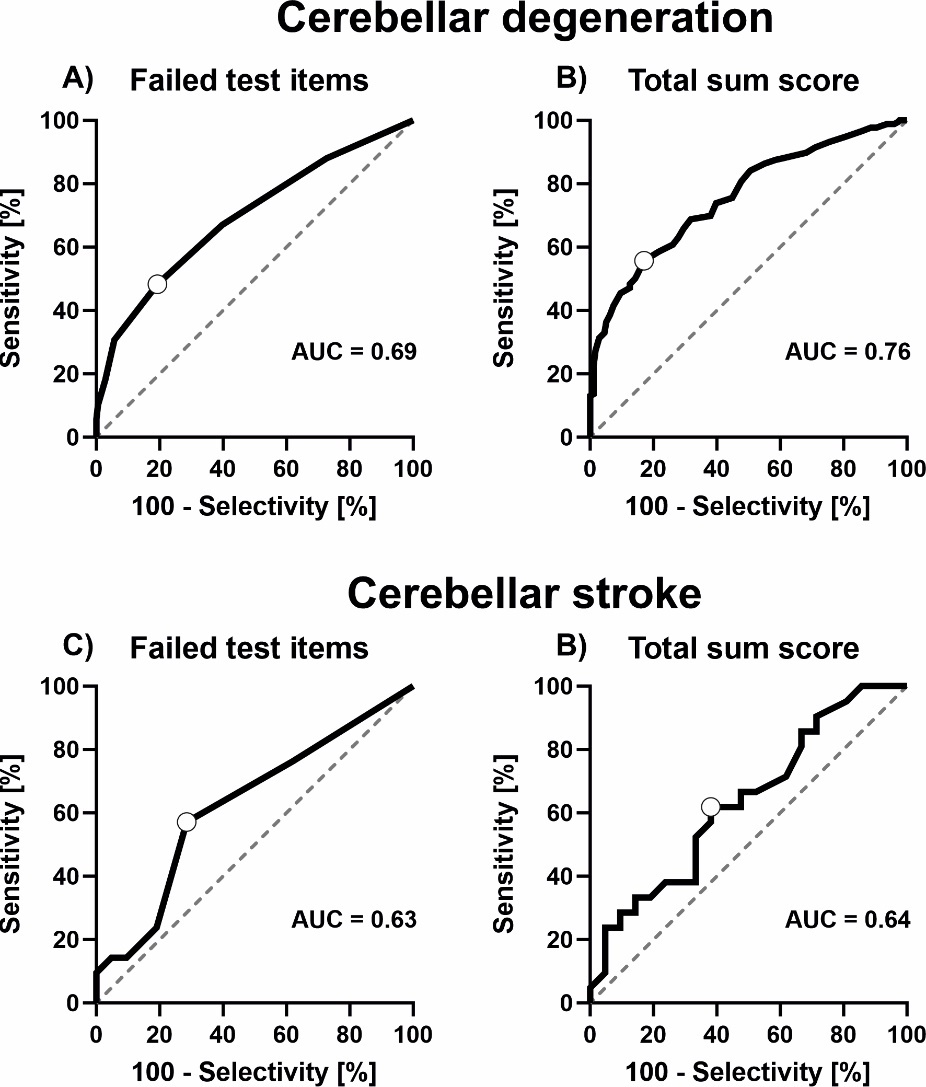


Receiver operating curves for the number of failed test items and the total sum raw score are shown for patients with cerebellar degeneration (A, B) and patients with cerebellar stroke (C, D). Clear circles indicate the Youden Index. AUC: area under the curve. Note, that according to previous literature, an AUC between 0.5 and 0.7 is considered poor, between 0.7 and 0.8 is good and an AUC of > 0.8 is excellent [1-3].

Regarding single test item raw scores, ROC analyses revealed good discriminative ability between patients with cerebellar degeneration and matched controls for *semantic fluency*, *phonematic fluency* and *category switching* (AUCs ≥ 0.7, p-values < 0.001). All other test items yielded poor ability to differentiate patients with cerebellar degeneration from matched controls (all AUC ≤ 0.66, p-values ≥ 0.001, Tab. S7, Fig. S2A) which conforms with the group comparisons for the *uncorrected* single items’ raw scores described in the main manuscript (see also Fig. S3A).

Patients with cerebellar stroke were well differentiated from matched controls on the *go/no-go* item (AUC ± SE = 0.77 ± 0.08, p <0.001). The remaining items had poor abilities to differentiate between patients with cerebellar stroke and controls (all AUC < 0.67, p-values ≥ 0.046) and some items (*digit span forward*, *cube drawing*, *verbal recall* and *similarities*) did not even exceed chance level (all AUC < 0.5, p-values ≥ 0.495, Tab. S7, Fig. S2B and S3B).

**Table S7: Area under (AUC) the receiver operating curves (ROC) for the single test items.**

| **Test item** | **Cer-deg (n = 176) vs.**  **Con-deg (n = 176)**  AUC ± SE  p-value | **Cer-str (n = 21) vs.**  **Con-str (n = 21)**  AUC ± SE  p-value |
| --- | --- | --- |
| **Semantic fluency** | **0.73 ± 0.03**  **p < 0.001*** | 0.59 ± 0.09  p = 0.310 |
| **Phonematic fluency** | **0.71 ± 0.03**  **p < 0.001*** | 0.61 ± 0.09  p = 0.198 |
| **Category switching** | **0.70 ± 0.03**  **p < 0.001*** | 0.53 ± 0.09  p = 0.747 |
| **Digit span forward** | **0.56 ± 0.03**  **p = 0.048*** | 0.44 ± 0.09  p = 0.495 |
| **Digit span backward** | **0.59 ± 0.03**  **p = 0.003*** | 0.53 ± 0.09  p = 0.708 |
| **Cube drawing** | **0.57 ± 0.03**  **p = 0.022*** | 0.47 ± 0.09  p = 0.774 |
| **Verbal recall** | 0.51 ± 0.03  p = 0.805 | 0.49 ± 0.09  p = 0.871 |
| **Similarities** | **0.59 ± 0.03**  **p = 0.004*** | 0.49 ± 0.09  p = 0.890 |
| **Go/no-go** | 0.55 ± 0.03  p = 0.083 | **0.77 ± 0.08**  **p < 0.001*** |
| **Affect** | **0.66 ± 0.03**  **p < 0.001*** | **0.67 ± 0.09**  **p = 0.046*** |
| **Overall Scores** | **Cer-deg (n = 176) vs.**  **Con-deg (n = 176)**  AUC ± SE  p-value  Youden Index (sensitivity/ selectivity) | **Cer-str (n = 21) vs.**  **Con-str (n = 21)**  AUC ± SE  p-value  Youden Index (sensitivity/ selectivity) |
| **Failed test items** | **0.69 ± 0.03**  **p < 0. 001***  **3 (48% / 81%)** | 0.63 ± 0.09  p = 0.146  2 (57% / 71%) |
| **Total Sum Score** | **0.76 ± 0.03**  **p < 0.001***  **90 (56%/ 83%)** | 0.64 ± 0.09  p = 0.116  97 (62%/ 62%) |

Statistics: Area under the receiver operating curve (AUC) ± standard error (SE) (raw score) is given for the single test items as well as the total number of failed test items and the total sum raw score. Additionally, the Youden index (that is the optimal cut-off weighting sensitivity and selectivity equally) with resulting values for sensitivity and selectivity in parenthesis is given for the total number of failed test items and the total sum raw score. Abbreviations: Cer-deg = patients with cerebellar degeneration, Con-deg = matched controls for Cer-deg, Cer-str = patients with focal lesions due to isolated cerebellar stroke, Con-str = matched controls for Cer-str, n = number.

**Figure S2: Receiver operating curves (ROC) for single test items.**


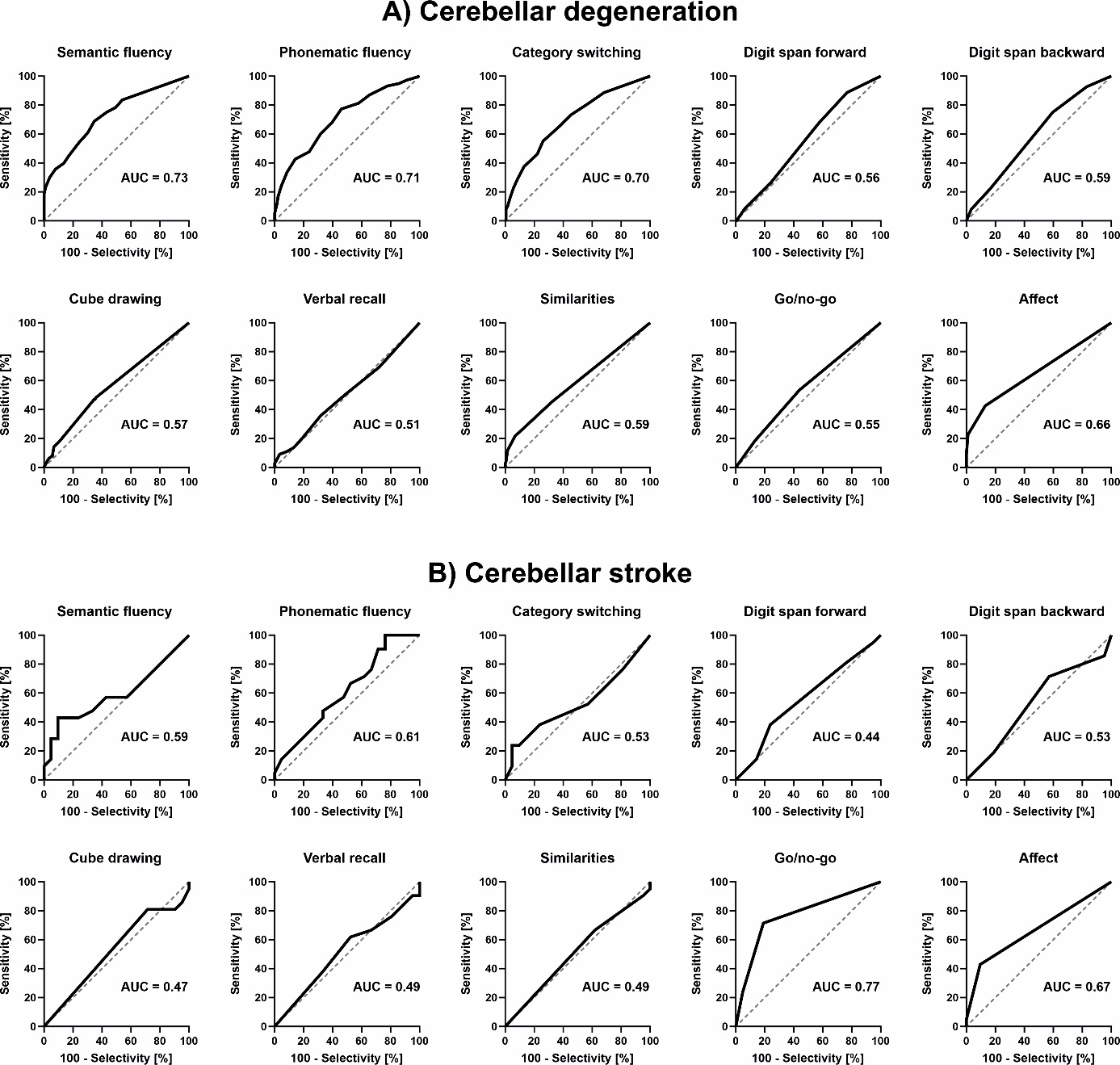


Receiver operating curves of the single test items are shown for patients with cerebellar degeneration (A) and patients with cerebellar stroke (B) versus matched controls. AUC: area under the curve. Note, that according to previous literature, an AUC between 0.5 and 0.7 is considered poor, between 0.7 and 0.8 is good and an AUC of > 0.8 is excellent [1-3].

**M3. Formulas for calculation of the standard error of measure and the minimal detectable change at the 80% and 95% confidence interval.**

Standard error of measure

SEM = SD_pooled_ * $\sqrt{1-ICC}$

*where SD_pooled_ is the arithmetic mean of the standard deviations of the 1^st^ and 2^nd^ test session*

Minimal detectable change at the 80% confidence interval

MDC80% = 1.28 * MDC *$\surd2$

*where MDC = SEM ** $\surd2$

Minimal detectable change at the 80% confidence interval

MDC95% = 1.96 * MDC *$\surd2$

*where MDC = SEM ** $\surd2$

**M4. Comparison of Cerebellar Cognitive Affective Syndrom Scale and Montreal Cognitive Assessment**

Methods

Performance on the German Cerebellar Cognitive Affective Syndrome Scale (G-CCAS-S; version A) and the German Montreal Cognitive Assessment (MoCA; version 1) were compared in a subgroup of 26 patients and 24 healthy controls. The total MoCA score was compared to the *uncorrected* total number of failed test items and the *uncorrected* total sum raw score of the G-CCAS. For statistical comparison the permutation t-test, described in the main manuscript, was used for the total sum raw score and for the total MoCA score and Fisher’s exact test was used for the number of failed test items. Sensitivity and selectivity were also compared.

Results

Patients and controls in this subanalysis did not differ regarding age (patients: mean ± standard deviation: 57.8 ± 9.8 yrs vs. controls: 61.6 ± 13.5 yrs; p = 0.280, two-sided permutation t-test), education (patients: 15.4 ± 2.8 yrs vs. controls 15.3 ± 2.7 yrs; p = 0.906, two-sided permutation t-test), or sex distribution (15/26 patients and 13/24 controls were male, p = 1.000, two-sided Fisher’s exact test).

Patients reached on average a similar total MoCA score as controls (patients: 26.5 ± 2.3 vs. controls: 27.5 ± 1.6 points), while they failed on average more *uncorrected* test items (patients: 2.0 ± 1.5 vs. controls: 1.3 ± 1.2) and had a lower *uncorrected* total sum raw score (patients: 92.5 ± 12.8 vs. controls: 99.3 ± 8.2) on the G-CCAS-S. The difference between patients and controls was significant for the total sum raw score of the G-CCAS-S [mean difference (MD), 95% confidence interval lower bound, upper bound: -6.8, -13.0, -0.9; p = 0.03), but failed significance for the total MoCA score (MD: -1.1, -2.3, -0.1; p = 0.072) and the number of failed test items of the G-CCAS-S (MD: 0.70, -0.1, 1.5; p = 0.07).

Importantly, both patients and controls performed on average within the normal range of the MoCA (≥ 26 points on the MoCA are considered normal test values). Six patients (23%) and three controls (12%) scored below 26 points on the MoCA. Of note, the three controls who scored slightly below 26 points (23 or 24 points) were 72, 81 and 84 years old and had 12 or 13 years of education. When applying our correction formula for the number of failed test items on the G-CCAS-S, 63% of the patients and 48% of the controls were considered *abnormal*. Using the correction for the total sum raw score of the G-CCAS-S, 42% of the patients and 0% of the controls were considered *abnormal*.

These results taken together indicate that the G-CCAS-S is (at least when considering the total sum raw score) more sensitive (i.e. more patients are correctly identified as patients) and selective (i.e. more controls are correctly identified as controls) than the MoCA.

**Table S8: Performance on single test items: Percentage of participants failing single test items and raw scores.**

| **Test item** | **Cer-deg (n = 176)** | **Con-deg (n = 176)** | **Cer-str (n = 21)** | **Con-str (n = 21)** |
| --- | --- | --- | --- | --- |
|  | **Failures on single test items** | | | |
| **Semantic fluency** | **23%** | **1%** | 10% | 0% |
|  | **p < 0.001*** | | p = 0.488 | |
| **Phonematic fluency** | **60%** | **32%** | 48% | 33% |
|  | **p < 0.001*** | | p = 0.530 | |
| **Category switching** | **47%** | **22%** | 33% | 19% |
|  | **p < 0.001*** | | p = 0.484 | |
| **Digit span forward** | 27% | 24% | 19% | 24% |
|  | p = 0.714 | | p = 1.000 | |
| **Digit span backward** | 22% | 17% | 19% | 19% |
|  | p = 0.224 | | p =1.000 | |
| **Cube drawing** | 18% | 11% | 19% | 10% |
|  | p = 0.068 | | p = 0.663 | |
| **Verbal recall** | 14% | 13% | 14% | 10% |
|  | p = 1.000 | | p = 1.000 | |
| **Similarities** | **22%** | **7%** | 10% | 5% |
|  | **p < 0.001*** | | p = 1.000 | |
| **Go/no-go** | 18% | 13% | 24% | 5% |
|  | p = 0.240 | | p = 0.184 | |
| **Affect** | **23%** | **1%** | 5% | 0% |
|  | **p < 0.001*** | | p = 1.000 | |
|  | | | | |
| **Test item** | **Cer-deg (n = 176)** | **Con-deg (n = 176)** | **Cer-str (n = 21)** | **Con-str (n = 21)** |
|  | **Raw scores on single test items** | | | |
| **Semantic fluency** | **19.5 ± 4.9** | **23.2 ± 3.3** | 21.6 ± 4.5 | 23.3 ± 3.0 |
|  | **MD: -3.7 [-4.6, -2.8]**  **p < 0.001*** | | MD: -1.7 [-4.1, 0.5]  p = 0.141 | |
| **Phonematic fluency** | **8.6 ± 4.1** | **11.6 ± 3.8** | 9.8 ± 4.0 | 11.8 ± 4.6 |
|  | **MD: -3.0 [-3.9, -2.2]**  **p < 0.001*** | | MD: -2.0 [-4.6, 0.5]  p = 0.136 | |
| **Category switching** | **9.6 ± 3.8** | **12.1 ± 3.0** | 11.2 ± 3.3 | 11.7 ± 2.5 |
|  | **MD: -2.5 [-3.2, -1.7]**  **p < 0.001*** | | MD: -0.5 [-2.2, 1.2]  p = 0.571 | |
| **Digit span forward** | **6.1 ± 1.1** | **6.3 ± 1.2** | 6.3 ± 1.1 | 6.1 ± 1.0 |
|  | **MD: -0.3 [-0.5, -0.03]**  **p = 0.028*** | | MD: 0.2 [-0.5, 0.8,]  p = 0.471 | |
| **Digit span backward** | **4.0 ± 1.0** | **4.4 ± 1.0** | 4.2 ± 1.0 | 4.2 ± 0.9 |
|  | **MD: -0.4 [-0.6, -0.1]**  **p < 0.001*** | | MD: -0.05 [-0.6, 0.5]  P = 0.753 | |
| **Cube drawing** | **13.0 ± 2.5** | **13.6 ± 2.1** | 13.6 ± 3.0 | 13.8 ± 2.1 |
|  | **MD: -0.6 [-1.1, -0.2]**  **p = 0.007*** | | MD: -0.2 [-1.9, 1.2]  p = 0.739 | |
| **Verbal recall** | 12.8 ± 2.5 | 13.0 ± 2.0 | 12.8 ± 3.0 | 13.2 ± 1.8 |
|  | MD: -0.2 [-0.7, 0.2]  p = 0.306 | | MD: -0.4 [-2.0, 1.0]  p = 0.591 | |
| **Similarities** | **7.1 ± 1.3** | **7.6 ± 0.7** | 7.5 ± 0.8 | 7.6 ± 0.6 |
|  | **MD: -0.5 [-0.7, -0.3]**  **p < 0.001*** | | MD: -0.05 [-0.6, 0.3]  p = 0.682 | |
| **Go/no-go** | **1.3 ± 0.8** | **1.4 ± 0.7** | **1.0 ± 0.7** | **1.8 ± 0.5** |
|  | **MD: -0.1 [-0-3, 0.006]**  **p = 0.045*** | | **MD: -0.7 [-1.1, -0.3]**  **p < 0.001*** | |
| **Affect** | **5.2 ± 1.2** | **5.9 ± 0.4** | **5.5 ± 0.6** | **5.9 ± 0.3** |
|  | **MD: -0.7 [-0.9, -0.5]**  **p < 0.001*** | | **MD: -0.4 [-0.7, -0.1]**  **p = 0.002*** | |

Statistics: Two-sided Fisher’s exact test was applied to compare the patient and control groups regarding the percentage of participants who failed on specific test items. For the comparison of raw scores of single test items a two-sided permutation t-test was applied. Significant results are indicated in bold font and by asterisks*.* Abbreviations: Cer-deg = patients with cerebellar degeneration, Con-deg = matched controls for Cer-deg, Cer-str = patients with focal lesions due to isolated cerebellar stroke, Con-str = matched controls for Cer-str, p = p-value, n = number, MD = unpaired mean difference [95% confidence interval lower bound, upper bound].

**Figure S3: Raw scores on single test items.**


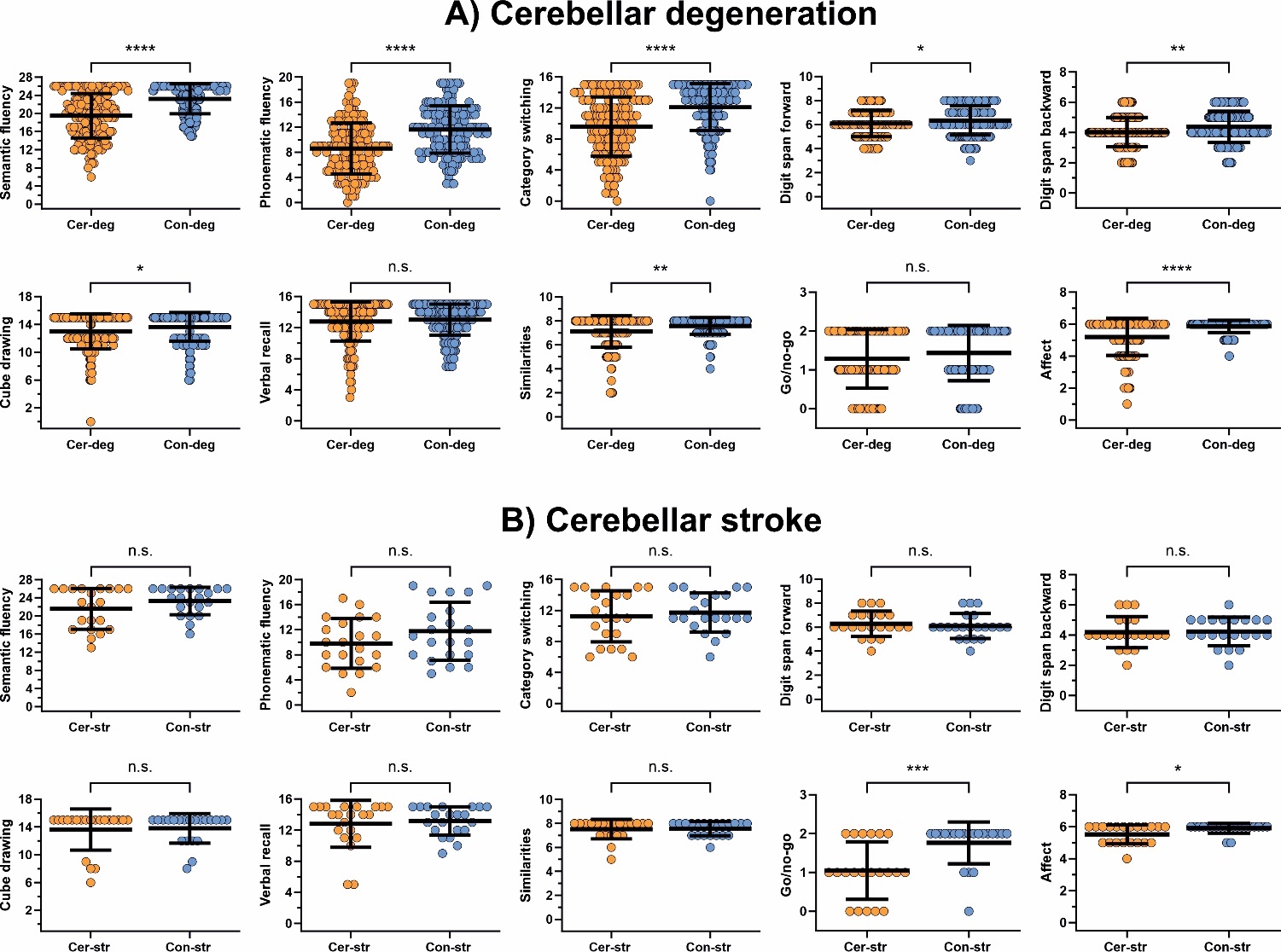


Raw scores on the single test items are shown for patients with cerebellar degeneration (orange; A) and patients with cerebellar stroke (orange; B) and their respective matched controls (blue; A-B). Each circle represents one participant. Error bars display means and standard deviations. Significant group comparisons are indicated by asterisks. Abbreviations: n.s. = not significant.

**Table S9: Overall *uncorrected* CCAS-S scores for test-retest (intrarater) and interrater reliability.**

| **Test-retest (intrarater) reliability** | **Patients** | | **Controls** | |
| --- | --- | --- | --- | --- |
|  | **T1** | **T2** | **T1** | **T2** |
| ***Uncorrected***  **failed test items** | 2.9 ± 1.7 | 2.5 ± 1.9 | 1.4 ± 1.6 | 0.9 ± 1.2 |
| ***Uncorrected***  **total sum score** | 87.5 ± 11.9 | 88.9 ± 12.9 | 100.3 ± 11.6 | 103.9 ± 10.5 |
| **Interrater**  **Reliability** | **Patients** | | **Controls** | |
|  | **T1** | **T2** | **T1** | **T2** |
| ***Uncorrected***  **failed test items** | 3.1 ± 2.5 | 3.2 ± 2.3 | 1.8 ± 1.7 | 1.1 ± 1.2 |
| ***Uncorrected***  **total sum score** | 86.1 ± 13.5 | 85.3 ± 16.7 | 99.4 ± 10.9 | 103.8 ± 8.8 |

Abbreviations: T1 = 1^st^ test session, T2 = 2^nd^ test session.

**Table S10: Correlations between test performance and time to retest.**

| **Measure** | **Patients** | | | **Controls** | | |
| --- | --- | --- | --- | --- | --- | --- |
|  | **Test-retest reliability** | **Interrater**  **reliability** | **Both subgroups** | **Test-retest reliability** | **Interrater**  **Reliability** | **Both subgroups** |
| **Δ *Uncorrected***  **failed test items** | r = 0.162  p = 0.483 | r = 0.014  p = 0.956 | r = 0.109  p = 0.502 | r = -0.188  p = 0.402 | r = -0.076  p = 0.731 | r = -0.111  p = 0.468 |
| **Δ *Uncorrected***  **total sum score** | r = -0.301  p = 0.185 | r = 0.017  p = 0.944 | r = -0.188  p = 0.246 | r = 0.232  p = 0.298 | r = 0.012  p = 0.957 | r = 0.131  p = 0.392 |

Statistics: Δ *uncorrected* failed test items = *uncorrected* failed test items at T2 - *uncorrected* failed test items at T1; Δ *uncorrected* total sum raw score = *uncorrected* total sum raw score at T2 - *uncorrected* total sum raw score at T1; R^2^ = Spearman’s rank correlation coefficient.

**References**

1. D'Agostino RB, Pencina MJ, Massaro JM, Coady S (2013) Cardiovascular Disease Risk Assessment: Insights from Framingham. Glob Heart 8:11-23

2. Faletti R, Battisti G, Discalzi A, Grognardi ML, Martinello S, Oderda M, Gontero P, Bergamasco L, Cassinis MC, Fonio P (2016) Can DW-MRI, with its ADC values, be a reliable predictor of biopsy outcome in patients with suspected prostate cancer? Abdom Radiol (NY) 41:926-933

3. Terwee CB, Bot SD, de Boer MR, van der Windt DA, Knol DL, Dekker J, Bouter LM, de Vet HC (2007) Quality criteria were proposed for measurement properties of health status questionnaires. J Clin Epidemiol 60:34-42

4. Thieme A, Roeske S, Faber J, Sulzer P, Minnerop M, Elben S, Jacobi H, Reetz K, Dogan I, Barkhoff M, Konczak J, Wondzinski E, Siebler M, Mueller O, Sure U, Schmahmann JD, Klockgether T, Synofzik M, Timmann D (2020) Validation of a German version of the Cerebellar Cognitive Affective/ Schmahmann Syndrome Scale: preliminary version and study protocol. Neurol Res Pract 2:39

5. Van der Schouw YT, Verbeek AL, Ruijs JH (1992) ROC curves for the initial assessment of new diagnostic tests. Fam Pract 9:506-511
